# Causal Associations Between Remnant Cholesterol Levels and Atherosclerosis-Related Cardiometabolic Risk Factors: A Bidirectional Mendelian Randomization Analysis

**DOI:** 10.3390/genes16020157

**Published:** 2025-01-26

**Authors:** Yu-Shien Ko, Lung-An Hsu, Semon Wu, Mei-Siou Liao, Ming-Sheng Teng, Hsin-Hua Chou, Yu-Lin Ko

**Affiliations:** 1 Department of Cardiology, Linkou Medical Center, Chang Gung Memorial Hospital, Taoyuan City 33305, Taiwan; c12037@cgmh.org.tw (Y.-S.K.); hsula@cgmh.org.tw (L.-A.H.); 2 College of Medicine, Chang Gung University, Taoyuan City 33305, Taiwan; 3 Department of Life Science, Chinese Culture University, Taipei 11114, Taiwan; semonwu@yahoo.com.tw; 4 Cardiovascular Center and Division of Cardiology, Department of Internal Medicine, Taipei Tzu Chi Hospital, Buddhist Tzu Chi Medical Foundation, New Taipei City 23142, Taiwan; d119098015@tmu.edu.tw (M.-S.L.); chouhhtw@gmail.com (H.-H.C.); 5 Department of Research, Taipei Tzu Chi Hospital, Buddhist Tzu Chi Medical Foundation, New Taipei City 23142, Taiwan; vincent@tzuchi.com.tw; 6 School of Medicine, Tzu Chi University, Hualien 97004, Taiwan

**Keywords:** remnant cholesterol, genome-wide association study, Mendelian randomization, cardiometabolic risk factor, diabetes mellitus, metabolic liver disease

## Abstract

**Background**: Despite the widespread use of lipid-lowering agents, the risk of atherosclerotic cardiovascular disease (ASCVD) remains; this residual risk has been attributed to remnant cholesterol (RC) levels. However, the causal associations between RC levels and various atherosclerosis-related cardiometabolic and vascular risk factors for ASCVD remain unclear. **Methods**: Using genetic and biochemical data of 108,876 Taiwan Biobank study participants, follow-up data of 31,790 participants, and follow-up imaging data of 18,614 participants, we conducted a genome-wide association study, a Functional Mapping and Annotation analysis, and bidirectional Mendelian randomization analyses to identify the genetic determinants of RC levels and the causal associations between RC levels and various cardiometabolic and vascular risk factors. **Results**: We found that higher RC levels were associated with higher prevalence or incidence of the analyzed risk factors. The genome-wide association study unveiled 61 lead genetic variants determining RC levels. The Functional Mapping and Annotation analysis revealed 21 gene sets exhibiting strong enrichment signals associated with lipid metabolism. Standard Mendelian randomization models adjusted for nonlipid variables and low-density lipoprotein cholesterol levels unraveled forward causal associations of RC levels with the prevalence of diabetes mellitus, hypertension, microalbuminuria, and metabolic liver disease. Reverse Mendelian randomization analysis revealed the causal association of diabetes mellitus with RC levels. **Conclusions**: RC levels, mainly influenced by genes associated with lipid metabolism, exhibit causal associations with various cardiometabolic risk factors, including diabetes mellitus, hypertension, microalbuminuria, and metabolic liver disease. This study provides further insights into the role of RC levels in predicting the residual risk of ASCVD.

## 1. Introduction

Remnant cholesterol (RC) is the cholesterol content of triglyceride-rich lipoproteins, such as chylomicrons in the non-fasting state and very-low-density lipoproteins and intermediate-density lipoproteins in the fasting state [1,2]. Accumulating evidence has highlighted the causal role of triglyceride-rich lipoproteins and their cholesterol-enriched remnants in the development and progression of atherosclerotic cardiovascular disease (ASCVD). Triglyceride-rich lipoproteins can act as a pro-inflammatory stimulus by releasing preformed mediators of oxidative stress with altered endothelial barrier function in vivo [3,4]. Chronic low-grade inflammation is considered as a common underlying pathophysiological feature of cardiometabolic diseases, such as obesity, diabetes mellitus, metabolic liver disease, and cardiovascular disorders [5,6]. Through Mendelian randomization (MR) studies, elevated RC has been shown to be causally associated with low-grade inflammation and the risk of ischemic heart disease [7,8]. RC levels are also associated with the occurrence of major adverse cardiovascular events in patients with ASCVD and the prevalence of cardiometabolic and vascular risk factors for ASCVD [8,9,10,11,12,13,14,15,16]. Despite the widespread use of statins, the risk of ASCVD persists, particularly in individuals with low levels of low-density lipoprotein cholesterol (LDL-C); this residual risk is ascribed to elevated RC levels under dyslipidemia conditions [17,18,19,20,21]. Thus, elucidating the causal associations between RC levels and atherosclerosis-related cardiometabolic and vascular risk factors for ASCVD can clarify whether RC can be targeted for reducing the residual risk of ASCVD [22].

Mendelian randomization (MR), a statistical approach wherein genetic variants are used as instrumental variables (IVs), can identify causal associations unaffected by confounders [23]. Recently, we identified a significant association between *APOB* variants and RC levels [24]. Several lipid-related gene variants have been used as IVs in the MR of RC levels to elucidate the causal associations of RC levels with C-reactive protein levels, 25-hydroxyvitamin D deficiency, ischemic heart disease, and aortic stenosis [7,8,25,26,27,28,29]. However, a genome-wide association study (GWAS) using summary statistics from the UK Biobank and FinnGen biobank database found no causal associations between RC levels and most of the analyzed cardiometabolic risk factors, except for lipid-related factors [15]. Thus, despite emerging evidence for strong associations of RC levels with various cardiometabolic and vascular risk factors [10,12,15,21,30,31], causality remains to be established.

Functional Mapping and Annotation (FUMA) is a web-based platform that incorporates multiple biological databases. It is widely used in genetic research for mapping and annotating genetic variants identified in GWASs [32]. In the present study, we used FUMA along with multivariate MR analysis for the purpose of analyzing data from the Taiwan Biobank (TWB) to elucidate the candidate genes and gene sets associated with RC levels. In addition, we analyzed the directions and magnitudes of the causal associations between genetically determined RC levels and various atherosclerosis-related cardiometabolic and vascular risk factors. This study provides valuable insights into the etiological associations between these variables, thereby laying the foundation for novel therapeutic interventions and preventive measures.

## 2. Materials and Methods

### 2.1. Study Cohort

**Figure 1:**
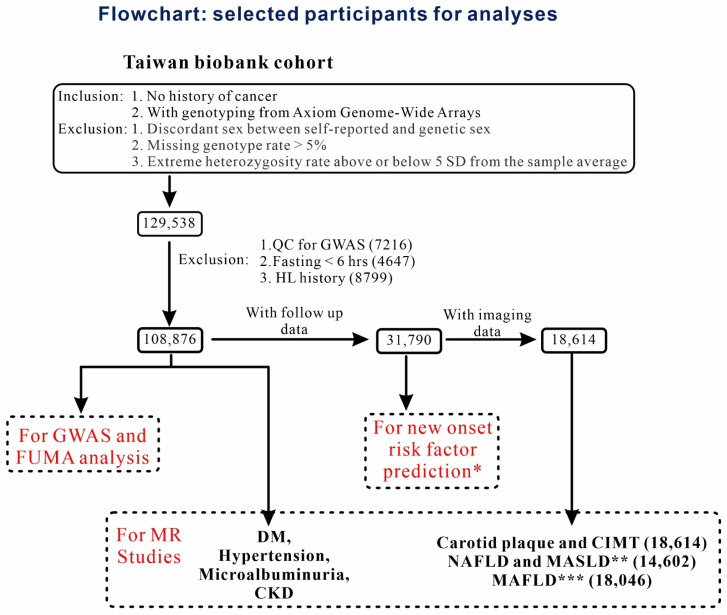
Participant selection for the analysis. For the present study, participants of the Taiwan Biobank study were screened against the inclusion and exclusion criteria. The results of quality control of GWAS data indicated the absence of imputation data and exhibited cryptic relatedness (identity-by-descent value > 0.187). * Exclusion of each cardiometabolic risk factor during the first survey. ** Exclusion of HBV infection, HCV infection, alcohol consumption, or other known causes of chronic liver disease (*n* = 4012). *** Exclusion of known causes of chronic liver disease (*n* = 568). Abbreviations: MR: Mendelian randomization; GWAS, genome-wide association study; HL, hyperlipidemia; DM, diabetes mellitus; CKD, chronic kidney disease; CIMT, carotid intimal-medial thickness; NAFLD, nonalcoholic fatty liver disease; MASLD, metabolic dysfunction–associated steatotic liver disease; MAFLD, metabolic dysfunction-associated fatty liver disease; SD: standard deviation.

TWB is an ongoing population-based prospective cohort study funded by the Taiwanese government; it includes Taiwan’s residents aged between 30 and 70 years. For the present study, we obtained Axiom Genome-Wide CHB Array data (from February 2008 to December 2020) of 129,538 TWB participants. After applying the exclusion criteria, we included 108,876 participants in the final cohort. In addition, we included 31,790 participants who had undergone follow-up for 4.2 ± 1.2 years (between July 2011 and November 2021) to investigate the associations between RC levels and new-onset cardiometabolic risk factors. Among these participants, 18,614 underwent additional abdominal and carotid Doppler ultrasound examinations. The ultrasound examinations focused on determining the carotid intima-media thickness (CIMT) and detecting carotid plaques and hepatic steatosis. Data from these participants were subjected to further association and MR analyses. Figure 1 presents a flowchart depicting the participant selection.

Supplementary Method S1 presents the definitions of various parameters used in the present study. This study was approved by the research ethics committees of Taipei Tzu Chi Hospital (permit number: 08-XD-005) and the Buddhist Tzu Chi Medical Foundation. The TWB study was approved by the Ethics and Governance Council of the TWB (permit number: TWBR11107-03). Written informed consent was obtained from all participants before the present study. All procedures were performed in accordance with the ethical principles outlined in the Declaration of Helsinki.

### 2.2. Clinical Characteristics and Laboratory Examinations

In addition to analyzing participants’ baseline characteristics, such as body shape indices and blood pressure, we explored their lipid profiles—levels of total cholesterol (TC), high-density lipoprotein cholesterol (HDL-C), and triglyceride. A colorimetric assay was performed using Hitachi LST008 (Hitachi, Naka, Japan) to evaluate glucose metabolism-related parameters (fasting plasma glucose and hemoglobin A1c levels) and serum creatinine levels. The level of RC was calculated by subtracting the cumulative level of HDL-C and LDL-C from that of TC. The details of LDL-C measurements are presented in Supplementary Method S2.

### 2.3. Genomic DNA Extraction, Genotyping, and GWAS

Genomic DNA was extracted from the participants’ whole blood samples. Genetic variants were genotyped using TWB chips and the Axiom Genome-Wide Array Plate system (Affymetrix, Santa Clara, CA, USA). Data were imputed using the SHAPEIT (version 2.12) and IMPUTE2 (version 2.3.2) software packages. Genetic variants were detected through GWAS and selected according to preset exclusion and inclusion criteria (Supplementary Method S3). GWAS data were analyzed through linear and logistic regression analyses.

### 2.4. Genomic Locus Definition and Functional Annotation

The GWAS results were used as input for the FUMA analysis. FUMA integrates information from biological databases and analytical tools for the functional annotation of genes and their variants, thereby identifying independent genomic loci [32]. Details of the FUMA analysis are presented in Supplementary Method S4.

### 2.5. Statistical Analysis

Continuous variables were logarithmically transformed and then subjected to a regression analysis. For MR analysis, the levels of TC, LDL-C, HDL-C, and RC were logarithmically transformed. Continuous data are presented in terms of median and interquartile range values, whereas categorical data are presented in terms of number and percentage values. Linear regression models were adjusted for age, sex, body mass index (BMI), and current smoking status to measure the effects of genetic variants on the continuous variables in the association studies. Bonferroni correction was performed for all MR analyses (*p* < 0.002, 0.05/11/2). Through logistic regression analysis, odds ratios and 95% confidence intervals were calculated to evaluate the effects of polymorphisms on the risks associated with categorical data. Furthermore, Cox regression analysis was performed to calculate hazard ratios for new-onset cardiometabolic risk factors. A *p* value of <5 × 10^−8^ indicated genome-wide significance. The PLINK software (version 1.07; https://zzz.bwh.harvard.edu/plink/; Shaun Purcell, Cambridge, MA, USA; accessed on 14 August 2021) was used for GWAS and conditional analyses. Linkage disequilibrium (LD) was examined using LDmatrix (version 5.6.8, https://analysistools.nci.nih.gov/LDlink/?tab=ldmatrix, accessed on 19 April 2021). All statistical analyses were performed using SPSS (version 22; IBM Corporation, Armonk, NY, USA). Missing data were approached with list-wise deletion.

### 2.6. MR Analysis

**Figure 2:**
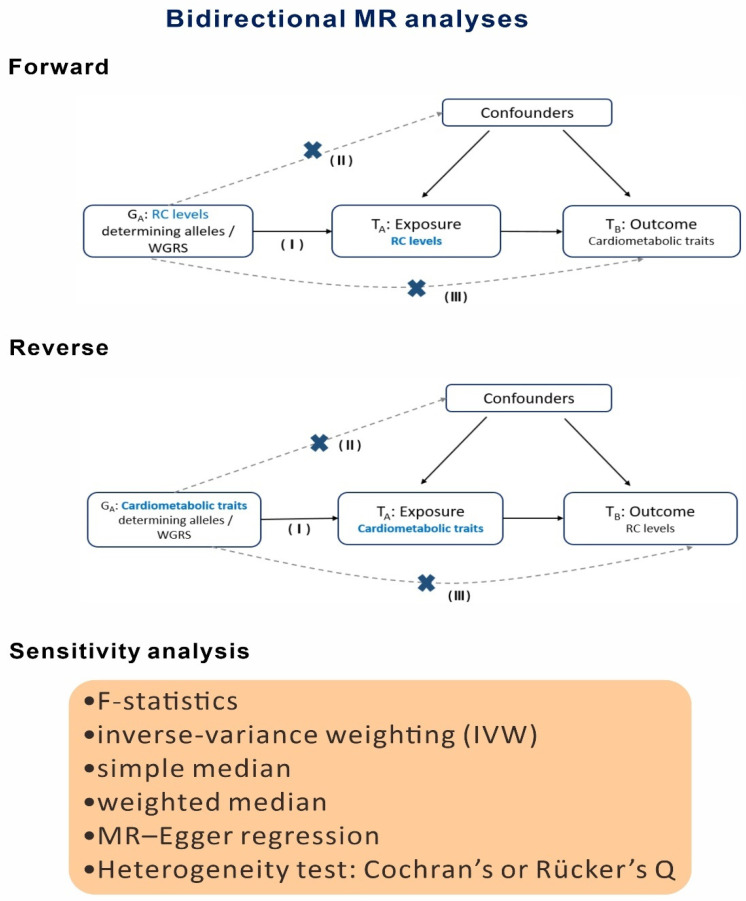
Diagrams for the approach of bidirectional MR analysis. The bidirectional MR analysis included the forward analysis (direction: from RC levels [exposure] to cardiometabolic and vascular risk factors [outcome]) and the reverse analysis (direction: from the risk factors [exposure] to RC levels [outcome]). The three core instrumental variable assumptions were relevance (I), independence (II), and exclusion restriction (III). Sensitivity analyses were performed to confirm the causal associations through MR analysis with multiple genetic variants.

Using the two-stage least squares (2SLS) method, we conducted a bidirectional one-sample MR analysis to identify the causal associations of RC levels with cardiometabolic, renal, hepatic, and vascular risk factors. The design of the MR analysis is depicted in Figure 2. In the forward MR analysis, the forward direction was from RC levels (exposure) to the aforementioned risk factors (outcomes). By contrast, in the reverse MR analysis, the reverse direction was from the aforementioned risk factors (exposure) to RC levels (outcome). Weighted genetic risk scores (WGRSs) were derived for the genetic variants determining RC levels (hereafter denoted as RC-WGRSs) and for each cardiometabolic and vascular risk factor; these scores were used as IVs in the MR analysis. In the first stage of the forward MR analysis, RC-WGRSs were regressed to predict RC levels. In the second stage of the analysis, the study parameters were regressed to predict the risk factors. In the first stage of the reverse MR analysis, the cardiometabolic and vascular risk factors determining the WGRSs were regressed to predict the RC levels. The GWAS results were subjected to quality control. The MR analysis included 108,876 participants and 3,640,178 variants. The lead variants that were significantly associated (*p* < 0.01) with each risk factor after adjustments for RC levels were excluded from the forward MR analysis, and those that were significantly associated (*p* < 0.01) with RC levels after adjustments for each risk factor were excluded from the reverse MR analysis. WGRSs were calculated by the sum of all the products of the risk alleles at each genetic locus and the effect sizes obtained from our GWASs. For reliable assessment of causal associations in the MR analysis, genetic variants were required to satisfy the following three IV assumptions: relevance: all genetic variants used for calculating WGRSs should be significantly associated with the exposure; independence: the associations between WGRSs and the outcomes of interest should be unaffected by confounders; and exclusion restriction: WGRSs should influence the outcomes of interest through exposure and not through any other pathways. To reduce bias in and ensure the validity of the MR analysis results, we addressed several potential limitations: we (1) determined the strength of IVs by using an F value of >10 to assess the limitation of weak bias in genetic variants, (2) excluded variants in significant LD, (3) minimized genotyping errors by assessing the Hardy–Weinberg equilibrium, and (4) evaluated potential unbalanced or directional horizontal pleiotropy through comprehensive sensitivity analyses [33]. The details are provided in Supplementary Methods S3 and S5.

### 2.7. Sensitivity Analysis

Various statistical approaches, such as inverse-variance weighting (IVW) with fixed effects and random effects, MR–Egger regression, simple median and weighted median value estimation, and funnel plot creation, were adopted to mitigate the influence of horizontal pleiotropy. Scatter plots, Cochran’s Q statistic for IVW, and Rücker’s Q statistic for MR–Egger regression were used to examine heterogeneity. The specific details are presented in Supplementary Method S5.

## 3. Results

### 3.1. Associations of RC Levels with Participants’ Clinical Characteristics and Laboratory Data

Supplementary Table S1 revealed the mean circulating RC levels of participants (21.91 ± 14.41 for mg/dL; 0.57 ± 0.14 for mmol/L). We explored the associations of RC levels with various clinical characteristics, biochemical markers, and metabolic risk factors in a sample of 108,876 participants from the TWB study (Table 1). After adjustments for age, sex, current smoking status, and BMI, the analysis revealed that RC levels were significantly associated with parameters such as body shape indices, blood pressure, lipid profile, glucose metabolism, and renal function (all *p* < 0.0001).

### 3.2. Associations of RC Levels with the Prevalence of Cardiometabolic and Vascular Risk Factors and New-Onset Cardiometabolic Risk Factors

**Figure 3:**
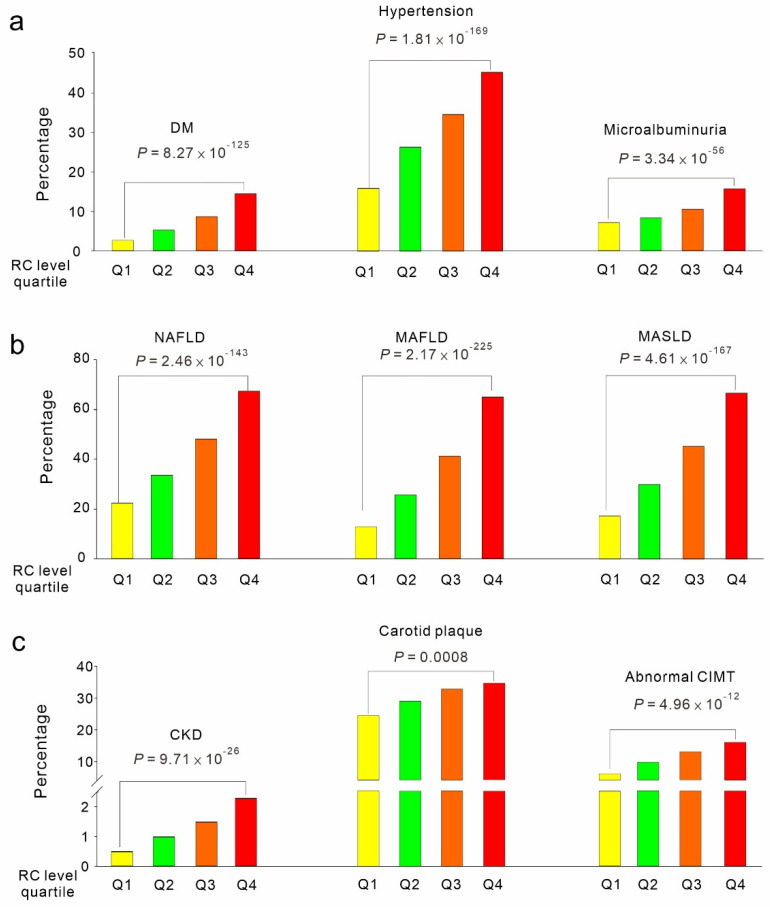
Associations of RC levels with various cardiometabolic and vascular risk factors. Cardiometabolic and vascular risk factors analyzed in the present study included prevalent conditions such as DM, hypertension, microalbuminuria, NAFLD, MAFLD, MASLD, CKD, carotid plaques, and abnormal CIMT. RC levels were categorized into four equal quartiles, designated Q1 to Q4, with Q4 representing the quartile with the highest numerical values (**a**–**c**). To determine significance, *p* values were obtained from statistical models adjusted for sex, age, body mass index, and current smoking status and calculated using logistic regression. Abbreviations: RC: remnant cholesterol; DM, diabetes mellitus; NAFLD, nonalcoholic fatty liver disease; MASLD, metabolic dysfunction-associated steatotic liver disease; MAFLD, metabolic dysfunction-associated fatty liver disease; CKD, chronic kidney disease; CIMT, carotid intimal–medial thickness.

**Figure 4:**
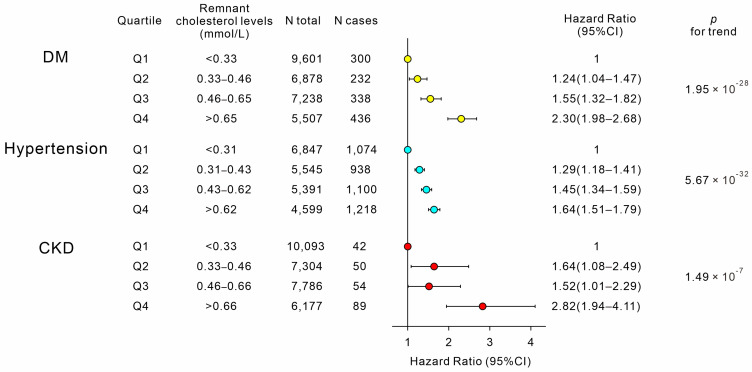
Associations between remnant cholesterol levels and new-onset cardiometabolic risk factors during the follow-up period. Abbreviations: DM, diabetes mellitus; CKD, chronic kidney disease.

RC levels were categorized into four quartiles (Q1–Q4) in ascending order. The increasing quartiles of RC levels were associated with an elevated prevalence of cardiometabolic risk factors, such as diabetes mellitus (DM), hypertension, chronic kidney disease (CKD), microalbuminuria, metabolic liver disease (nonalcoholic fatty liver disease [NAFLD], metabolic dysfunction-associated fatty liver disease [MAFLD], and metabolic dysfunction-associated steatotic liver disease [MASLD]), as well as vascular risk factors, such as carotid plaques and abnormal CIMT (Figure 3a–c). With the increasing quartiles of RC levels, participants who initially had no cardiometabolic risk factors during the first survey exhibited increased risks of new-onset DM, hypertension, and CKD during the follow-up period (Figure 4).

### 3.3. GWAS of the Associations of Genetic Variants with RC Levels and Cardiometabolic and Vascular Risk Factors

A GWAS was conducted to identify the associations of genetic variants with RC levels and cardiometabolic and vascular risk factors (Supplementary Figures S1 and S2; Supplementary Tables S2–S4). After adjustments for age, sex, smoking status, and BMI, the analysis revealed 61 lead variants that were significantly associated with RC levels (all *p* < 5 × 10^−8^). The F value exceeded 14 for all variants, except for rs2914225. Notably, the rs7350481 variant exhibited the strongest association with the highest RC level (*p* < 1.0 × 10^−307^). The rs7350481 variant is located near the *BUD13*/*APOA5*/*APOA4*/*APOC3*/*APOA1* gene cluster region on chromosome 11q23.3 (Supplementary Figure S1; Supplementary Table S2). For DM, hypertension, and microalbuminuria, the analysis revealed 16, 22, and 2 lead variants, respectively, after adjustments for age, sex, smoking status, and BMI (all F values > 10; Supplementary Figure S2A–C; Supplementary Table S3). After the exclusion of genetic variants on the basis of the exclusion criteria, significant associations were noted between all metabolic liver disease-determining genetic variants and RC levels after further adjustments for NAFLD, MAFLD, and MASLD (*p* < 0.01). Thus, these genetic variants were not included in the reverse MR analysis. Consequently, we could not construct a reverse MR analysis model adjusted for NAFLD, MAFLD, and MASLD (Supplementary Figure S2D–F; Supplementary Table S4). Furthermore, no significant genome-wide associations were noted between genetic variants and CKD, carotid plaque, or CIMT; thus, the reverse MR analysis could not be performed (Supplementary Figure S2G–I). The F values for the lead variants indicated a low probability of weak instrument bias. LD examinations revealed independent associations between lead variants located within 1 Mb of each other (Supplementary Table S5).

### 3.4. Summary of GWAS and FUMA Results

The detailed results of the FUMA analysis are presented in Supplementary Figures S3–S5 and Supplementary Tables S6–S12. In brief, 138 lead genetic variants were categorized into 59 genomic risk loci by merging LD blocks. A total of 541 lead genes in these loci were identified to be the genetic determinants of RC levels; these results were obtained through functional gene mapping approaches, such as positional mapping, expression quantitative trait locus mapping, and chromatin interaction mapping. High expression levels were observed for 134 genes in the liver. By characterizing the genomic loci, two genomic loci were mapped to two single-protein-coding genes—*ACACB* and *TCHP* in genomic loci 42 and 43, respectively; the results indicated that both genes contributed to their associations with RC levels (Supplementary Table S10). Furthermore, an analysis performed using the Multimarker Analysis of GenoMic Annotation tool revealed 21 gene sets associated with lipid metabolism; these genes exhibited strong enrichment signals, and they were involved in the following mechanisms: the assembly, remodeling, and clearance of plasma lipoproteins; the efflux, transport, and storage of sterol, cholesterol, and phospholipids in addition to the reverse transport of cholesterol; the formation of chylomicrons and triglyceride-rich lipoproteins; and the assembly and regulation of lipoproteins and hepatic lipases (Supplementary Table S12).

### 3.5. Results of Stepwise Linear Regression

A stepwise linear regression analysis was performed to identify the primary independent variables associated with RC levels. The results revealed significant associations of RC levels with age, sex, body shape indices, mean blood pressure, RC-WGRS, fasting plasma glucose level, renal function, and lifestyle habits (all *p* < 0.0001; Table 2).

### 3.6. Results of Bidirectional MR Analysis and 2SLS

**Figure 5:**
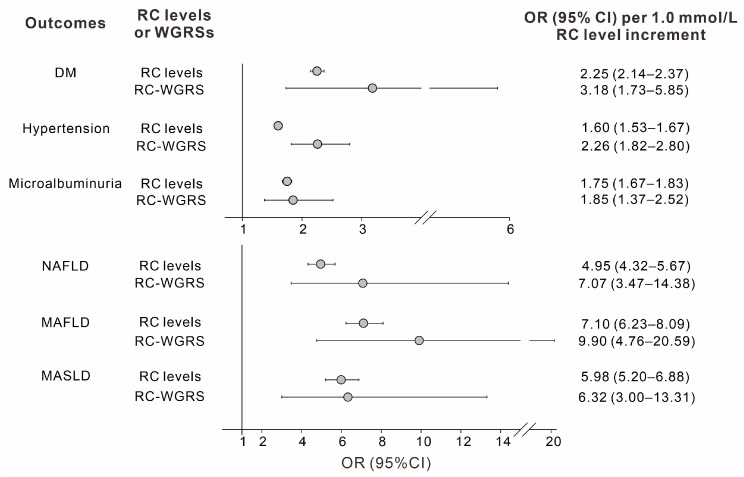
Observational and causal associations between remnant cholesterol (RC) levels and cardiometabolic outcomes. The odds ratios (ORs) showed the effects of RC levels (observational association), in comparison with the weighted genetic risk scores of RC levels (RC-WGRSs) (causal association), on the risk of cardiometabolic outcomes according to a standard increment of 1.0 mmol/L RC levels (all *p* < 0.0001). Corresponding 95% confidence interval (CI) values indicated the precision of the ORs. To determine significance, *p* values were obtained from logistic regression models adjusted for age, sex, body mass index, and current smoking status. The abbreviations of outcomes are shown in Figure 2.

The forward MR analysis involving 47–56 genetic variants for RC levels revealed that RC levels were significantly associated with DM, hypertension, microalbuminuria, NAFLD, MAFLD, and MASLD. After adjustments for age, sex, BMI, smoking status, nonlipid variables, and LDL-C levels, the 2SLS analysis revealed that RC-WGRSs were significantly associated with cardiometabolic risk factors. With further adjustment for RC levels, all the associations became nonsignificant after Bonferroni correction (*p* ≥ 0.002), indicating the causality of RC levels for most cardiometabolic risk factors (Table 3). The MR analysis using RC-WGRSs suggested that an increment of 1 mmol/L RC level was strongly associated with increasing risks of DM, hypertension, microalbuminuria, NAFLD, MAFLD, and MASLD (ORs: 3.18, 2.26, 1.85, 7.07, 9.90, and 6.32, respectively; all *p* values < 0.0001; Figure 5). When compared to non-MR (observational) analysis, causal associations with MR using RC-WGRSs showed at least a trend of higher ORs for cardiometabolic outcomes (Figure 5). By contrast, RC-WGRSs exhibited no significant association with CKD, carotid plaques, or abnormal CIMT (Table 3).

The reverse MR analysis indicated that a higher risk of DM (from a genetic perspective) was associated with higher RC levels. However, hypertension and microalbuminuria exhibited no significant reverse causal associations with RC levels (Table 3).

### 3.7. Results of Sensitivity Analysis

Sensitivity analyses further confirmed the causal associations between RC levels and cardiometabolic risk factors. The associations were identified through the forward MR analysis performed using the 2SLS method. Effect sizes were consistent across statistical approaches such as IVW, simple and weighted median value estimation, and MR–Egger regression (slope; Supplementary Figure S6). Corresponding funnel plots were symmetrical, suggesting the absence of directional pleiotropy effects across all sets of IV estimates and IV strength (Supplementary Figure S7). Scatter plots suggested that the associations between alleles determining RC levels and the risk factors were dependent on RC levels (Supplementary Figure S8). In the reverse MR analysis, sensitivity analyses also yielded consistent results. No significant directional pleiotropy was observed, which supported the causal effects of DM on RC levels (Supplementary Figures S9–S11). Cochran’s Q and Rücker’s Q tests revealed significant global heterogeneity (*p* < 0.05), suggesting that multiple pathways mediate the associations between IVs, RC levels, and the study outcomes, expect for MAFLD (Supplementary Table S13).

## 4. Discussion

In this study, we performed a GWAS, a FUMA analysis, and a bidirectional MR analysis to elucidate the causal associations of RC levels with various cardiometabolic, renal, hepatic, and vascular risk factors. Our findings indicated that RC levels were independently associated with the prevalence and incidence of risk factors such as adiposity, blood pressure, glucose metabolism, renal function, and lifestyle habits, and with conditions such as metabolic liver disease; terms such as NAFLD, MAFDL, or MASLD; carotid plaques; and abnormal CIMT. The results for functional annotation revealed strong enrichment signals for 21 gene sets associated with lipid metabolism. Furthermore, the forward MR analysis indicated significant causal associations between RC levels and conditions such as DM, hypertension, microalbuminuria, and metabolic liver disease. Bidirectional associations were observed between RC levels and DM, whereas no causal associations between RC levels and chronic kidney disease or vascular conditions were found. This study provides insights into the role of RC levels in predicting the residual risk of ASCVD.

In this study, we set the significance level for the associations between genetic variants and RC levels to *p* < 5 × 10^−8^. The GWAS revealed 61 lead genetic variants. Other GWASs have identified 30–54 lead variants [15,34,35]. Comparisons of the present and previous studies indicated similarities in the mapped genes. In our previous MR analyses of candidate genes associated with RC levels, we observed genome-wide significant associations for the variants of six genes: *APOA5*, *LPL*, *GCKR*, *TRIB1*, *ANGPTL3*, and *ANGPTL4*. Furthermore, *APOB*, *LPL*, *TIMD4*, *FADS2*, *LIPC*, *CETP*, and *APOC1-APOE-TOMM40* exhibited associations with RC levels in the present and previous studies [7,8,27]. We further performed functional annotation using FUMA to identify RC level-determining candidate genes. On the basis of the FUMA results, we mapped two genomic loci to the single-protein-coding genes *ACACB* and *TCHP*; the results indicated both genes as candidate genes. Acetyl-CoA carboxylase 2, which is encoded by *ACACB*, serves a regulator of fatty acid oxidation in mitochondria. *ACACB* knockout increased insulin sensitivity, mitigated high-fat-diet-induced obesity risks, and ameliorated hyperglycemia in mice [36,37]. Trichoplein keratin filament-binding protein, which is encoded by *TCHP*, partly colocalizes within the mitochondria. *TCHP* expression is often downregulated in patients with epithelial cancer [38]. *TCHP* knockout mitigated high-fat-diet-induced obesity risks and inhibited adipogenesis in mice [39]. Comprehensive genetic studies have demonstrated the associations of *ACACB* with HDL-C and triglyceride levels and the association of *TCHP* with HDL-C levels [40,41,42,43]. In the present study, a gene set enrichment analysis revealed strong enrichment signals for 21 gene sets associated with lipid metabolism at various RC levels. Together, these findings indicate that the genes associated with lipid metabolism are the major determinants of RC levels.

MR studies have uncovered key metabolic pathways associated with triglyceride-rich lipoproteins and their remnants, thereby clarifying the associated risks of cardiovascular, metabolic, and inflammatory disorders. Studies conducted before 2021 have predominantly adopted a candidate gene approach to explore atherogenic dyslipidemia. A major breakthrough was made in a collaborative analysis of 101 studies, in which a causal association between triglyceride levels and coronary artery disease was found on the basis of the rs662799 polymorphism in the promoter of *APOA5* [28]. In the context of lipid metabolism–related pathways, the presence of RC-increasing alleles imply that elevated nonfasting RC levels are causal risk factors for ischemic heart disease and myocardial infarction [7,8,27]. Si, et al. [29] analyzed 144 variants across 15 lipid profiles to explore the causal roles of circulating RC levels in cardiovascular and cerebrovascular diseases. Through a GWAS of up to 49 lead variants, causal associations were established between RC levels and various cardiovascular diseases, such as ischemic heart disease, myocardial infarction, peripheral artery disease, atrial fibrillation or flutter, and nonrheumatic valvular disease; however, the results obtained for cerebrovascular diseases remain debatable [15,35]. Recently, Wu et al. [44] further suggested that the RC level is causally associated with the risk of large-artery atherosclerosis stroke, but not other subtypes of ischemic stroke.

The associations of RC levels with cardiometabolic and vascular risk factors have been less commonly elucidated in MR studies. Guan et al. [15] selected 54 candidate variants from a GWAS and conducted a two-sample multivariate MR analysis by using data from predominantly European individuals. The researchers observed significant causal associations of elevated RC levels with increased prevalence of hypercholesterolemia and increased levels of TC, triglyceride, and LDL-C in both univariate and multivariate IVW models. However, they found no significant causal associations of RC levels with DM, hypertension, obesity, CKD, or NAFLD. Population studies have indicated specific associations between RC levels and new-onset DM, but no causal associations have been established [12,45]. In the present study, significant bidirectional causal associations were noted between RC levels and DM. In addition, we noted forward causal associations of RC levels with hypertension, microalbuminuria, and metabolic liver disease. These causal associations persisted even after adjustments for nonlipid variables and LDL-C levels. Emerging evidence from population-based cohort studies indicates that, compared with the recent alterations in the diagnostic criteria for NAFLD, changes in those for MAFLD and MASLD do not significantly affect disease prevalence or associated mortality [46,47,48]. We found that RC levels were causally associated with various forms of metabolic liver disease, irrespective of the diagnostic criteria. These findings confirm the key role of RC levels in predicting the risks of cardiometabolic and metabolic liver diseases and, thus, the residual risk of ASCVD.

Our results differ from those of Guan et al. [15], and several limitations should be mentioned. By using the same database to extract both exposure and outcome data, we analyzed all selected genetic variants. However, unlike Guan et al. [15], who used summary data for analysis, we could not effectively minimize the potential bias in causal evaluations. When calculating the WGRSs, we excluded target outcome-related variants, thereby minimizing the effect of horizontal pleiotropy. Our study participants were mostly Han Chinese in origin, and with genetic differences in ethnicity, the effects of genetic heterogeneity should be considered. In the present study, RC levels were calculated from other lipid variables; thus, we could not select variants that were completely independent of those associated with other lipids. Furthermore, we did not perform a comprehensive analysis of cardiovascular outcomes. This limitation should be addressed in future studies.

## 5. Conclusions

We conducted a GWAS with a FUMA analysis to identify lipid metabolism-associated gene sets that serve as the major genetic determinants of RC levels. Our MR analysis revealed forward or bidirectional causal associations of RC levels with diseases prevalent in the Taiwanese population—for example, DM, hypertension, microalbuminuria, and metabolic liver disease (NAFLD, MAFLD, and MASLD). This study clarifies the causal associations of RC levels with atherosclerosis-related cardiometabolic risk factors and provides insights into the role of RC levels in predicting the residual risk of ASCVD.

## Figures and Tables

**Table 1:** Association between remnant cholesterol levels and cardiometabolic and vascular traits and lifestyle habits in Taiwan Biobank participants.

		*N*	Remnant Cholesterol Levels (mmol/L)	*p* Value
Sex	Male	38,423	0.56 (0.39–0.82)	3.16 × 10^−299^
	Female	70,329	0.43 (0.31–0.62)	
Current smoking	No	87,969	0.45 (0.32–0.65)	1.52 × 10^−97^
	Yes	20,783	0.57 (0.39–0.84)	
Hypertension	No	75,673	0.43 (0.31–0.62)	1.51 × 10^−199^
	Yes	33,203	0.58 (0.41–0.83)	
Diabetes mellitus	No	100,236	0.46 (0.33–0.67)	5.82 × 10^−264^
	Yes	8516	0.66 (0.47–0.95)	
Metabolic syndrome	No	86,731	0.42 (0.31–0.57)	<10^−307^
	Yes	22,021	0.86 (0.62–1.13)	
Alcohol drinking	No	102,549	0.47 (0.33–0.68)	4.70 × 10^−30^
	Yes	6203	0.57 (0.39–0.88)	
Exercise	No	66,017	0.47 (0.33–0.70)	2.32 × 10^−107^
	Yes	42,735	0.47 (0.33–0.67)	
Microalbuminuria	No	97,222	0.46 (0.33–0.67)	2.11 × 10^−112^
	Yes	11,476	0.57 (0.38–0.86)	
NAFLD	No	8345	0.40 (0.30–0.55)	1.51 × 10^−149^
	Yes	6257	0.58 (0.41–0.84)	
MAFLD	No	10,375	0.40 (0.30–0.55)	5.87 × 10^−241^
	Yes	7671	0.63 (0.45–0.89)	
MASLD	No	8804	0.40 (0.29–0.55)	1.82 × 10^−175^
	Yes	5798	0.61 (0.43–0.86)	
CKD	No	107,424	0.47 (0.33–0.69)	4.08 × 10^−38^
	Yes	1450	0.63 (0.44–0.92)	
Carotid plaque	No	13,036	0.45 (0.33–0.65)	9.30 × 10^−5^
	Yes	5578	0.05 (0.36–0.72)	
Abnormal CIMT	No	13,635	0.45 (0.32–0.64)	1.91 × 10^−9^
	Yes	4979	0.53 (0.39–0.76)	

Based on the definitions of cardiometabolic and vascular traits and lifestyle habits (i.e., current smoking, drinking, exercise), the participants were divided into two groups, No and Yes, to assess the association between remnant cholesterol levels and these traits and habits. Remnant cholesterol levels represent adjustment for sex, age, body mass index, and current smoking. Sex represents adjustment for age, BMI, and current smoking. Shortened forms: NAFLD: nonalcoholic fatty liver disease; MAFLD: metabolic dysfunction-associated fatty liver disease; MASLD: metabolic dysfunction-associated steatotic liver disease; CKD: chronic kidney disease; and CIMT: carotid intimal–medial thickness.

**Table 2:** Stepwise linear regression analysis of RC levels in the Taiwan Biobank participants.

	β	se	r^2^	*p* Value
Waist–hip ratio	0.6685	0.0138	0.1677	<10^−307^
Body mass index	0.0126	0.0002	0.0428	<10^−307^
Mean BP	0.0021	0.0001	0.0188	2.05 × 10^−211^
RC-WGRS	0.9311	0.0213	0.018	<10^−307^
Fasting plasma glucose	0.0013	0	0.0114	1.34 × 10^−231^
eGFR	−0.0007	0	0.0062	1.25 × 10^−93^
Current smoking	0.0302	0.0021	0.0035	6.25 × 10^−47^
Exercise	−0.0267	0.0016	0.002	5.55 × 10^−65^
Age	0.0009	0.0001	0.001	1.15 × 10^−27^
Alcohol drinking	0.024	0.0032	0.0006	1.20 × 10^−13^
Sex	−0.0066	0.0019	0.0001	5.49 × 10^−4^

The parameters were ranked from high to low on the basis of the coefficients (r^2^) of their correlations with RC levels. Abbreviations: BP: blood pressure; eGFR, estimated glomerular filtration rate; RC, remnant cholesterol; RC-WGRS, weighted genetic risk score for RC.

**Table 3:** Summary of correlation coefficients used in the bidirectional Mendelian randomization analysis.

T_A_	T_B_	G_A_ **	T_A_–T_B_			G_A_–T_A_			G_A_–T_B_			IV_A_–T_B_				
			β	SE	*p ^a^*	β	SE	*p ^a^*	β	SE	*p ^a^*	β	SE	*p ^a^*	*p ^b^*	*p ^c^*
RC	DM ^#^	47 *	1.893	0.055	5.82 × 10^−264^	0.968	0.022	<10^−307^	1.558	0.421	2.0 × 10^−4^	1.610	0.435	2.0 × 10^−4^	0.002	0.392
DM	RC ^#^	16 ^†^	0.070	0.002	4.56 × 10^−180^	1.027	0.033	3.7 × 10^−213^	0.009	0.002	1.6 × 10^−7^	0.009	0.002	1.6 × 10^−7^	1.2 × 10^−5^	0.026
RC	HTN ^#^	53 *	1.065	0.035	1.51 × 10^−199^	0.986	0.013	<10^−307^	1.197	0.158	3.7 × 10^−14^	1.213	0.160	3.7 × 10^−14^	5.3 × 10^−11^	0.236
HTN	RC ^#^	22 ^†^	0.040	0.002	7.81 × 10^−149^	0.903	0.027	1.7 × 10^−239^	−0.001	0.002	0.812	−0.001	0.003	0.812	0.845	0.041
RC	MA ^#^	53 *	1.041	0.046	2.11 × 10^−112^	0.989	0.014	<10^−307^	0.890	0.225	7.8 × 10^−5^	0.899	0.228	7.8 × 10^−5^	8.9 × 10^−5^	0.264
MA	RC ^#^	2 ^†^	0.044	0.002	4.24 × 10^−99^	1.003	0.124	6.2 × 10^−16^	0.011	0.008	0.163	0.011	0.008	0.163	0.175	0.253
RC	NAFLD ^##^	56 *	2.480	0.095	1.51 × 10^−149^	0.973	0.016	<10^−307^	2.530	0.457	3.2 × 10^−8^	2.600	0.470	3.2 × 10^−8^	6.6 × 10^−7^	0.551
RC	MAFLD ^###^	52 *	3.177	0.096	5.87 × 10^−241^	0.977	0.017	<10^−307^	2.997	0.485	6.5 × 10^−10^	3.068	0.497	6.5 × 10^−10^	2.0 × 10^−8^	0.593
RC	MASLD ^##^	53 *	2.827	0.100	1.82 × 10^−175^	0.973	0.016	<10^−307^	2.466	0.482	3.1 × 10^−7^	2.535	0.495	3.1 × 10^−7^	5.0 × 10^−6^	0.877
RC	CKD ^#^	58 *	1.577	0.122	4.08 × 10^−38^	0.986	0.013	<10^−307^	0.276	0.583	0.636	0.279	0.591	0.636	--	--
RC	Carotid plaque ^####^	61 *	0.321	0.082	9.30 × 10^−5^	0.922	0.028	3.5 × 10^−226^	0.147	0.331	0.656	0.160	0.359	0.656	--	--
RC	Abnormal CIMT ^####^	59 *	0.550	0.092	1.91 × 10^−9^	0.983	0.013	<10^−307^	0.288	0.390	0.460	0.031	0.424	0.460	--	--

* WGRSs of 47–61 RC level–determining significant variants in the GWAS. ^†^ WGRSs of 2–22 DM-, hypertension (HTN)-, and microalbuminuria (MA)-determining significant variants in the GWAS. *^a^* Adjusted for age, sex, smoking, and body mass index. *^b^* Further adjusted for low-density lipoprotein cholesterol level and possible confounders such as WHR, MBP, eGFR, fasting plasma glucose level, albuminuria, alcohol consumption, and exercise habits. (*p ^b^*: DM: WHR, MBP, eGFR, albuminuria, alcohol consumption, and exercise habits; HTN: WHR, eGFR, fasting plasma glucose level, albuminuria, alcohol consumption, and exercise habits; MA: WHR, MBP, eGFR, fasting plasma glucose level, alcohol consumption, and exercise habits). *^c^* Further adjusted for RC levels, DM, HTN, or MA. **: Number of lead SNPs in WGRS. Number of participants: #: 108,876, ##: 14,602, ###: 18,046, ####: 186,614. Abbreviations: IV_A_: Instrumental variables for G_A_; RC, remnant cholesterol; GWAS, genome-wide association study; WGRS, weighted genetic risk score; DM, diabetes mellitus; NAFLD, nonalcoholic fatty liver disease; MASLD, metabolic dysfunction-associated steatotic liver disease; MAFLD, metabolic dysfunction-associated fatty liver disease; CKD, chronic kidney disease; CIMT, carotid intimal–medial thickness; WHR, waist-to-hip ratio; MBP, mean blood pressure; eGFR, estimated glomerular filtration rate.

## Data Availability

The original contributions presented in this study are included in the article/Supplementary Materials. Further inquiries can be directed to the corresponding author.

## Supplementary Materials

The following supporting information can be downloaded at: https://www.mdpi.com/article/10.3390/genes16020157/s1, Supplementary Methods S1–S5. Figure S1: A genome-wide association study for remnant cholesterol (RC) levels in Taiwan Biobank participants; Figure S2: Genome-wide association studies for cardiometabolic and vascular traits in Taiwan Biobank participants; Figure S3: Summary results for a genome-wide association study of the remnant cholesterol (RC) levels with FUMA; Figure S4: Overview of prioritized genes from a genome-wide association study of the remnant cholesterol (RC) levels with FUMA; Figure S5: The chromatin interaction and eQTLs of genomic risk loci for remnant cholesterol (RC) levels on chromosomes; Figure S6: Estimates of the causal links between RC-WGRSs and cardiometabolic traits by standard MR methods; Figure S7: Hypothetical funnel plots of the causal effect estimate between RC-WGRSs and cardiometabolic traits; Figure S8: Scatter plots of putative causal relationships between RC-determining alleles on cardiometabolic traits; Figure S9: Estimates of the causal links between cardiometabolic traits-WGRSs and remnant cholesterol (RC) levels in standard MR sensitivity analysis; Figure S10: Hypothetical funnel plots of the causal effect estimate between cardiometabolic traits-WGRSs and remnant cholesterol (RC) levels; Figure S11: Scatter plots of putative causal relationships between RC-determining alleles on cardiometabolic traits; Table S1: The mean circulating remnant cholesterol levels of Taiwan biobank participants; Table S2: Lead variants in a genome-wide association study for remnant cholesterol (RC) levels; Table S3: Lead variants in genome-wide association studies for various cardiometabolic traits; Table S4: The lead variants in genome-wide association studies for metabolic liver disease and their association with each trait and remnant cholesterol (RC) level without (column of RC level) or with (column of Trait_adj_RC level) adjustment of each trait; Table S5: The linkage disequilibrium (LD) test between remnant cholesterol level determining lead variants within 1 Mb of the chromosomal region; Table S6: Independent significant SNPs (r^2^ < 0.6) identified from remnant cholesterol level GWAS; Table S7: Lead SNPs identified from independent significant SNPs of remnant cholesterol level GWAS; Table S8: Genomic risk loci of interest from remnant cholesterol level GWAS; Table S9: Expression values of prioritized genes in 30 tissues; Table S10: Prioritized genes from remnant cholesterol level GWAS by functional mapping.; Table S11: Enriched gene sets identified by prioritized genes from remnant cholesterol level GWAS; Table S12: magma.gsa.sets.genes; Table S13: Cochran’s Q and Rücker’s Q tests for heterogeneity.

## Abbreviations

2SLS	Two-stage least squares
ASCVD	Atherosclerotic cardiovascular disease
BMI	Body mass index
CIMT	Carotid intima–media thickness
CKD	Chronic kidney disease
DM	Diabetes mellitus
FUMA	Functional mapping and annotation
GWAS	genome-wide association study
HDL-C	High-density lipoprotein cholesterol
IVs	Instrumental variables
IVW	Inverse-variance weighting
LD	Linkage disequilibrium
LDL-C	Low-density lipoprotein cholesterol
MAFLD	Metabolic dysfunction-associated fatty liver disease
MASLD	Metabolic dysfunction-associated steatotic liver disease
MR	Mendelian randomization
NAFLD	Nonalcoholic fatty liver disease
RC	Remnant cholesterol
TC	Total cholesterol
TWB	Taiwan Biobank
WGRSs	Weighted genetic risk scores
