# Supplementary material for: Causal Associations Between Remnant Cholesterol Levels and Atherosclerosis-Related Cardiometabolic Risk Factors: A Bidirectional Mendelian Randomization Analysis
Source: Genes (Basel). 2025 Jan 26;16(2):157. doi: 10.3390/genes16020157 (PMC11855473; doi:10.3390/genes16020157)
Supplement: Supplementary file 1 [file genes-16-00157-s001.zip › Supplementary_Figures and Tables.pdf]

Causal associations between remnant cholesterol levels and atherosclerosis-related cardiometabolic risk factors: A bidirectional Mendelian randomization analysis

Supplementary Table S1. The mean circulating remnant cholesterol levels of Taiwan biobank participants.

	Mean ± SD
Remnant cholesterol (mg/dL)	21.91 ± 14.41
Remnant cholesterol (mmol/L)	0.57 ± 0.14

The numbers of participants were 108,876. SD: standard deviation.

Supplementary Figure S1. A genome-wide association study for remnant cholesterol (RC) levels in Taiwan Biobank participants.

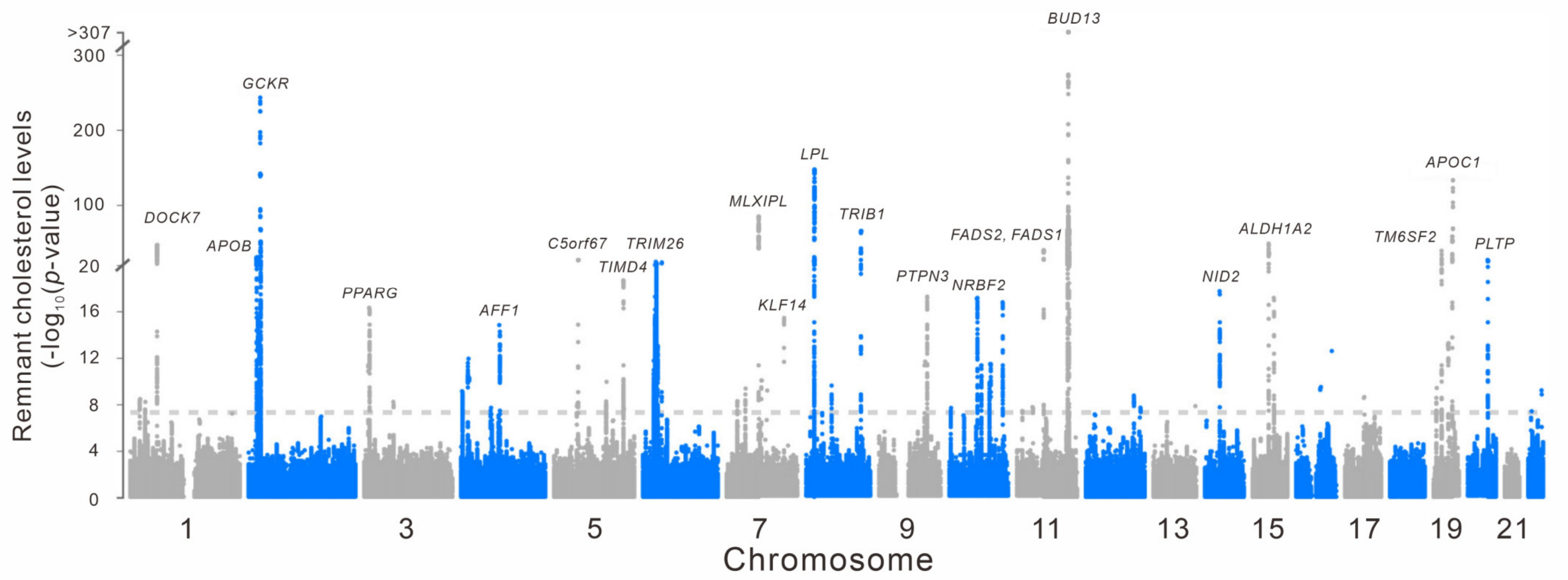

Manhattan plot displayed the genome wide significant gene loci for RC levels in Taiwanese population. -log 10 (*p* value) adjusted for age, sex, current smoking status and body mass index.

Supplementary Figure S2. Genome-wide association studies for cardiometabolic and vascular traits in Taiwan Biobank participants.

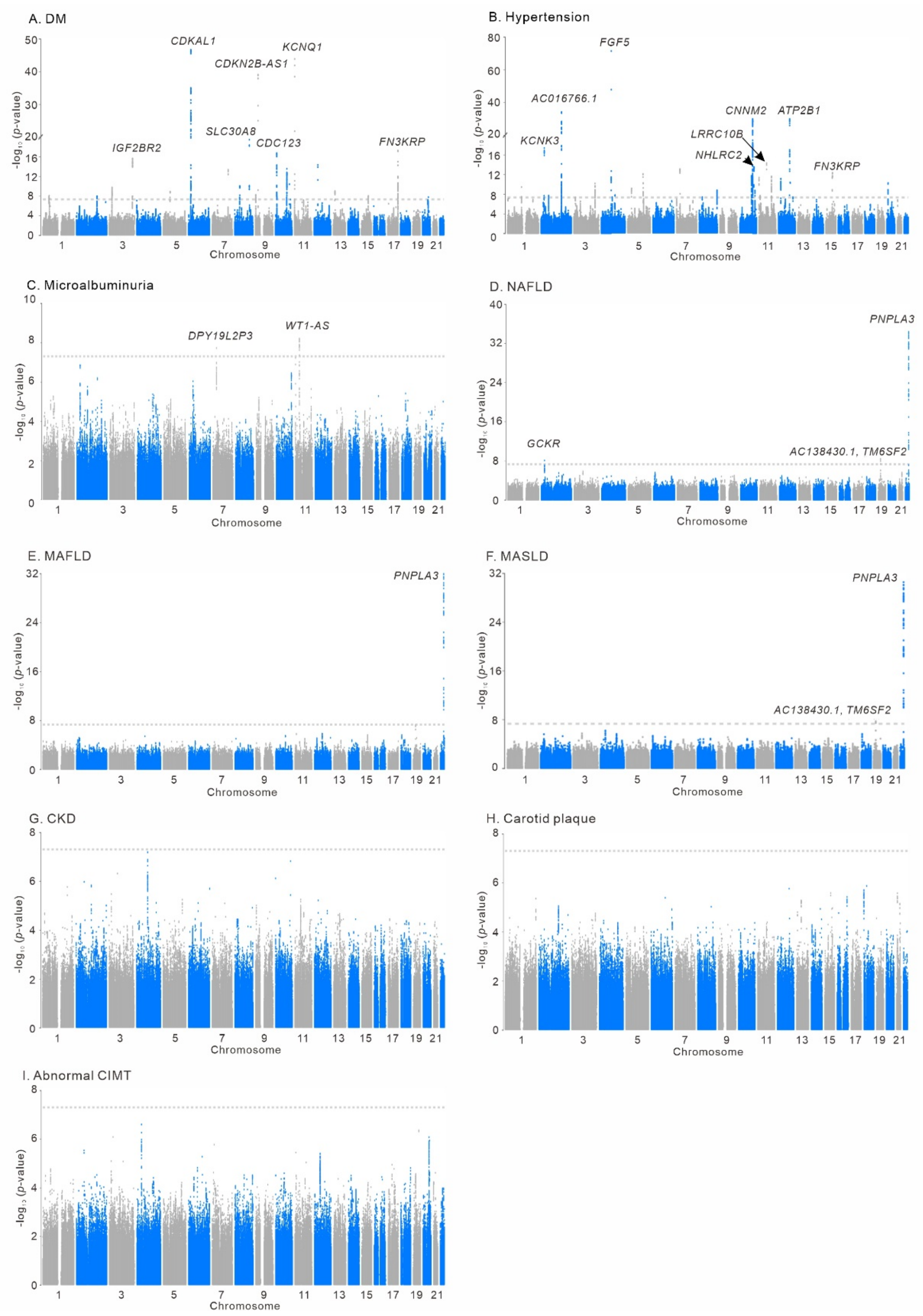

Manhattan plots displayed the genome-wide significant gene loci for each cardiometabolic and vascular

traits in Taiwanese population. -log 10 (*p* value) was adjusted for age, sex, current smoking status and body mass index.

Supplementary Table S2. Lead variants in a genome-wide association study for remnant cholesterol (RC) levels

							RC levels						
CHR	SNP	BP	genes	Ref/Alt	MAF	HWE	BETA	SE	L95	U95	*P*	F-statistics	R^2
11	rs7350481	116715567	*BUD13*	C/ T	0.2374	0.315	0.0529	0.001	0.05083	0.05492	0.00E+00	2028.050109	0.018278
2	rs6547692	27512105	*GCKR*	A/ G	0.492	0.3354	0.0297	0.0009	0.02797	0.03145	1.28E-243	813.657354	0.007449
8	rs6586884	20005847	*LPL*	T/ C	0.1011	0.6842	-0.0381	0.0015	-0.041	-0.0353	8.72E-148	527.613110	0.004815
19	rs438811	44913484	*AC011481.3*	C/ T	0.1746	0.9292	0.0289	0.0012	0.02663	0.03123	1.02E-133	508.053582	0.004647
7	rs3812316	73606007	*MLXIPL*	C/ G	0.0859	0.8849	-0.031	0.0016	-0.0342	-0.0279	3.28E-85	295.539427	0.002698
8	rs2001846	125466208	*AC091114.1*	C/ T	0.4369	0.7771	0.0154	0.0009	0.01368	0.0172	4.35E-66	223.837412	0.002043
15	rs1532085	58391167	*ALDH1A2*	G/ A	0.4407	0.5682	0.0133	0.0009	0.01152	0.01505	2.46E-49	173.938917	0.001586
1	rs10789113	62549528	*DOCK7*	A/ T	0.2092	0.1564	-0.0158	0.0011	-0.018	-0.0137	2.46E-47	175.108821	0.001598
11	rs174560	61814292	*FADS2, FADS1*	C/ T	0.4214	0.0095	-0.012	0.0009	-0.0138	-0.0103	1.42E-40	112.959194	0.001028
19	rs58542926	19268740	*AC138430.1, TM6SF2*	C/ T	0.06618	0.6291	-0.0236	0.0018	-0.0271	-0.0201	7.22E-40	140.784583	0.001283
2	rs35131127	20983042	*AC115619.1*	T/ C	0.08703	0.9145	0.0185	0.0016	0.0154	0.02162	2.03E-31	105.641386	0.000962
20	rs148753678	45907567	*PLTP*	CAA/ -	0.06675	0.5506	0.0196	0.0018	0.01606	0.02311	1.35E-27	94.041022	0.000865
5	rs465002	56512648	*C5orf67*	T/ C	0.4694	0.1535	-0.0097	0.0009	-0.0115	-0.008	1.44E-27	94.451276	0.000859
8	rs1041983	18400285	*NAT2*	C/ T	0.4323	0.6938	-0.0095	0.0009	-0.0112	-0.0077	6.25E-26	74.650538	0.000676
6	rs117565607	30204594	*TRIM26*	T/ A	0.1114	0.6551	-0.0147	0.0014	-0.0175	-0.0119	3.96E-25	65.738208	0.000599
6	rs1358980	43796814	*AL157371.2*	T/ C	0.4486	0.7872	-0.0091	0.0009	-0.0109	-0.0074	2.75E-24	77.357536	0.000701
5	rs6882076	156963286	*TIMD4*	C/ T	0.2713	0.2033	-0.0091	0.001	-0.011	-0.0071	2.05E-19	68.607861	0.000621
14	rs79823890	52045251	*NID2*	G/ T	0.1186	0.734	-0.0121	0.0014	-0.0148	-0.0094	1.89E-18	25.304873	0.000223
9	rs77824033	109478856	*PTPN3*	T/ C	0.2296	0.3956	-0.0093	0.0011	-0.0114	-0.0072	5.55E-18	50.984513	0.000474
15	rs77648222	69890512	*DRAIC*	A/ G	0.2346	0.6979	-0.0091	0.0011	-0.0111	-0.007	7.04E-18	36.601980	0.000328
10	rs1396964	63118538	*AL590502.1*	A/ G	0.3876	0.6873	0.0079	0.0009	0.0061	0.00971	8.12E-18	73.470839	0.000670
10	rs2263985	121143992	*RPL19P16*	T/ C	0.4113	0.5373	-0.0077	0.0009	-0.0095	-0.0059	1.69E-17	45.661903	0.000411
3	rs7649970	12350773	*PPARG*	C/ T	0.03979	0.05768	-0.0191	0.0023	-0.0235	-0.0146	4.99E-17	42.254261	0.000379
7	rs13240528	130760369	*KLF14*	C/ A	0.31	0.1613	-0.0079	0.001	-0.0098	-0.006	3.76E-16	28.337651	0.000253
4	rs10023050	87143279	*AFF1*	A/ G	0.4101	0.559	-0.0073	0.0009	-0.0091	-0.0055	1.53E-15	57.174189	0.000521
19	rs1672991	35065755	*HPN, HPN-AS1*	G/ A	0.1596	0.4968	-0.0092	0.0012	-0.0116	-0.0068	5.70E-14	47.621110	0.000430
19	rs1423065	33359589	*AKR1B1P7*	C/ T	0.1724	0.625	0.0088	0.0012	0.00652	0.01116	8.21E-14	42.228783	0.000380
6	rs6909732	34251233	*AL354740.1*	C/ T	0.1035	0.8781	-0.0109	0.0015	-0.0138	-0.008	9.24E-14	24.330587	0.000215
16	rs2925979	81501185	*CMIP*	C/ T	0.4292	0.1979	0.0066	0.0009	0.00482	0.00835	2.62E-13	38.885030	0.000348
4	rs11941723	17810905	*NCAPG*	A/ G	0.1433	0.9353	0.0091	0.0013	0.00657	0.01158	1.20E-12	37.535892	0.000337
6	rs77681092	27016984	*LINC00240*	T/ -	0.1041	0.749	0.0103	0.0015	0.00746	0.01319	1.68E-12	16.299003	0.000141
9	rs2575876	104903458	*ABCA1*	G/ A	0.2309	0.02455	-0.0074	0.0011	-0.0095	-0.0053	2.12E-12	39.154447	0.000350
10	rs79117755	93023917	*EXOC6*	-/ C	0.1818	0.8697	0.0081	0.0012	0.00585	0.01043	3.46E-12	41.170085	0.000376
10	rs111567932	72904785	*OIT3*	T/ -	0.2891	0.9669	0.0068	0.001	0.00487	0.00872	4.77E-12	28.188027	0.000250
7	rs572295823	80661109	*CD36*	CA/ -	0.01959	1	-0.0208	0.0032	-0.027	-0.0145	8.97E-11	42.635905	0.000384

5	rs56017758	119404998	*TNFAIP8*	G/ A	0.1488	0.651	-0.0081	0.0013	-0.0106	-0.0057	1.17E-10	34.181233	0.000310
8	rs112784971	58493931	*CYP7A1*	C/ T	0.2304	0.8087	-0.0067	0.0011	-0.0088	-0.0047	2.46E-10	27.886395	0.000250
16	rs12149545	56959249	*CETP*	G/ A	0.1611	0.8582	-0.0076	0.0012	-0.01	-0.0053	3.27E-10	29.962185	0.000266
19	rs60748247	8435690	*MARCHF2*	-/ ACCACACCTGGCTAGTT	0.01353	0.1339	-0.0241	0.0038	-0.0316	-0.0165	3.97E-10	36.770505	0.000329
7	rs12534104	44180143	*GCK*	C/ A	0.226	0.06464	-0.0068	0.0011	-0.0089	-0.0046	4.39E-10	38.945321	0.000359
22	rs7291040	46089093	*MIRLET7BHG*	T/ C	0.1186	0.4999	-0.0087	0.0014	-0.0114	-0.0059	6.26E-10	22.715452	0.000205
7	rs445	92779056	*CDK6*	C/ T	0.3633	0.3633	0.0057	0.0009	0.00391	0.00755	6.55E-10	18.598019	0.000162
4	rs2748447	3336104	*RGS12*	A/ G	0.3517	0.07934	-0.0058	0.0009	-0.0076	-0.0039	7.59E-10	24.089937	0.000214
12	rs10744776	109255001	*ACACB*	G/ A	0.2507	0.1637	-0.0062	0.001	-0.0083	-0.0042	1.76E-09	30.597274	0.000275
17	rs929786922	44518409	*GPATCH8*	G/ A	0.01085	0.06031	0.0257	0.0043	0.01729	0.03419	2.37E-09	23.518149	0.000208
1	rs10917386	23472508	*ASAP3*	T/ C	0.4165	0.1367	-0.0053	0.0009	-0.0071	-0.0036	3.54E-09	22.500899	0.000198
7	rs4722564	26007843	*MIR148A*	G/ T	0.4665	0.602	0.0052	0.0009	0.00348	0.007	5.53E-09	28.482548	0.000254
3	rs73091851	66716141	*AC098969.2*	A/ G	0.1971	0.8847	0.0066	0.0011	0.00436	0.0088	6.19E-09	28.985524	0.000262
1	rs77968776	35235523	*ZMYM4*	A/ G	0.4433	0.8148	0.0053	0.0009	0.00352	0.00711	6.20E-09	19.538341	0.000176
12	rs75584653	110021680	*ANKRD13A*	C/ T	0.08946	0.9162	0.0091	0.0016	0.00603	0.01219	6.97E-09	26.747533	0.000239
5	rs11443126	53980126	*ARL15*	A/ -	0.1273	0.949	-0.0077	0.0013	-0.0103	-0.0051	8.77E-09	20.659199	0.000181
13	rs187712349	113548938	*TMCO3*	G/ A	0.0423	0.4392	0.0126	0.0022	0.00826	0.01698	1.41E-08	32.783173	0.000293
11	rs11820100	36285791	*COMMD9*	C/ T	0.1896	0.4536	-0.0064	0.0011	-0.0087	-0.0042	1.72E-08	19.521072	0.000170
12	rs10846601	124100606	*RFLNA*	C/ T	0.2543	0.4918	-0.0058	0.001	-0.0078	-0.0038	1.94E-08	25.565828	0.000229
4	rs9994887	68671757	*UGT2B15*	G/ A	0.4227	0.01109	-0.0051	0.0009	-0.0069	-0.0033	1.98E-08	21.323674	0.000188
6	rs55880953	33157187	*COL11A2*	G/ A	0.09812	0.1774	0.0084	0.0015	0.00549	0.01139	2.02E-08	14.379295	0.000123
10	rs75413156	5221531	*AKR1C4*	C/ A	0.1006	0.7531	-0.0083	0.0015	-0.0112	-0.0054	2.04E-08	18.515422	0.000161
5	rs2914225	158572051	*AC091939.1*	T/ C	0.3198	0.06609	0.0053	0.001	0.00345	0.0072	2.51E-08	9.593558	0.000079
11	rs6486122	13339977	*ARNTL*	C/ T	0.3675	0.7412	0.0051	0.0009	0.00328	0.00691	3.76E-08	37.010831	0.000331
22	rs5760102	23906574	*AP000350.6*	C/ T	0.4913	0.1629	0.0049	0.0009	0.00318	0.0067	3.98E-08	22.597924	0.000201
11	rs7936331	13812845	*LINC02548*	G/ A	0.488	0.7156	0.0049	0.0009	0.00315	0.00665	4.13E-08	32.005385	0.000285

GWAS analysis detected a total 61 lead SNPs associated with RC levels after adjustment for age, sex, BMI, and current smoking status. The rs7350481 was observed in highest peak ($p < 1.0 \times 10^{-307}$) and occur within *BUD13* on chromosome 11. *P* value was calculated by linear regression. F-statistic > 10 was used to check the strength of association between genetic instrumental variable and RC levels. R^2 indicated the proportion of variance in the exposure phenotype that is accounted for by the SNPs. Shortened forms: BETA: beta coefficient; BP: base position; CHR: chromosome; HWE: Hardy-Weinberg equilibrium; MAF: minor allele frequency; Ref/Alt: reference/alternate alleles; SE: standard error; SNP: single-nucleotide polymorphisms. L95: Lower bound of 95% confidence interval for odds ratio; H95: Upper bound of 95% confidence interval for odds ratio.

Supplementary Table S3. Lead variants in genome-wide association studies for various cardiometabolic traits.

Traits	CHR	SNP	BP	gene	ref/alt	MAF	HWE	traits			F-statistics	R^2
								beta	SE	*P*		
DM												
	6	rs138420022	20693466	*CDKAL1*	AT/A	0.3453	0.3891	0.2492	0.0172	2.27E-47	170.450161	0.001555
	9	rs10811661	22134095	*CDKN2B-AS1*	T/C	0.4158	0.3433	-0.2288	0.0173	9.02E-40	120.968350	0.001101
	17	rs1046875	82727550	*FN3KRP*	A/G	0.4846	0.3085	-0.145	0.0168	5.82E-18	73.647932	0.000667
	10	rs7896600	12213176	*CDC123*	C/G	0.4416	0.1006	-0.1438	0.0169	1.93E-17	63.928584	0.000578
	3	rs9854769	185803160	*IGF2BP2*	A/G	0.2495	0.9334	0.1553	0.0189	2.39E-16	55.207374	0.000499
	10	rs1112718	92719350	*Y_RNA*	G/A	0.1398	0.6877	0.1756	0.0231	2.97E-14	48.438201	0.000436
	7	rs7778167	128211575	*MIR129-1*	G/A	0.1261	0.9688	0.1832	0.0243	4.61E-14	50.320169	0.000464
	10	rs4506565	112996282	*TCF7L2*	A/T	0.02336	0.5333	0.3286	0.0499	4.19E-11	40.152659	0.000359
	8	rs890220	41620842	*GPAT4, AC009630.3*	G/A	0.1394	0.3105	-0.1643	0.0254	9.73E-11	41.757937	0.000380
	3	rs9882514	23252912	*UBE2E2*	A/G	0.3778	0.4425	-0.1109	0.0174	1.98E-10	36.761016	0.000329
	13	rs488166	32980214	*TOMM22P3*	G/C	0.2176	0.2155	0.1213	0.0199	1.02E-09	23.512526	0.000208
	9	rs4237150	4290085	*GLIS3*	G/C	0.4486	0.3462	0.0971	0.0168	6.28E-09	22.424063	0.000197
	1	rs59524047	50933543	*FAF1*	G/A	0.1004	0.1656	-0.1671	0.0291	8.88E-09	26.580059	0.000236
	15	rs34591043	77421244	*HMG20A*	A/G	0.3499	0.7878	0.0989	0.0173	9.91E-09	32.747938	0.000293
	2	rs3791352	164502516	*GRB14*	A/C	0.04821	0.2384	-0.2403	0.0422	1.23E-08	28.123227	0.000250
	20	rs34724263	47801576	*SULF2*	AG/A	0.1872	0.625	0.1187	0.021	1.84E-08	27.145827	0.000243
Hypertension												
	2	rs73029563	164151656	*AC016766.1*	C/G	0.5685	0.991	-0.1323	0.0107	7.97E-35	117.328819	0.001067
	12	rs111478946	89665065	*ATP2B1*	G/A	0.3158	0.1009	-0.1315	0.0114	1.39E-30	111.582820	0.001015
	10	rs11191548	103086421	*CNNM2*	T/C	0.2928	0.2859	-0.1338	0.0116	1.43E-30	71.916143	0.000651
	2	rs35021474	26693976	*KCNK3*	C/G	0.2559	0.2995	-0.1056	0.0122	4.26E-18	68.583636	0.000620
	11	rs10897165	61510413	*LRRC10B*	G/A	0.4148	0.5869	-0.0843	0.0108	5.07E-15	46.168492	0.000415
	10	rs11196569	113968393	*NHLRC2*	G/A	0.1376	0.3319	0.1151	0.0151	3.20E-14	46.035976	0.000413
	7	rs13243033	27208754	*HOTTIP*	T/C	0.6186	0.2187	-0.0819	0.011	8.03E-14	31.205487	0.000277
	15	rs1378940	74791153	*CSK*	C/A	0.1759	0.3262	-0.1009	0.0139	4.51E-13	50.633589	0.000456
	10	rs7072099	94276473	*PLCE1*	A/G	0.3316	0.3185	-0.079	0.0112	1.88E-12	28.785223	0.000255
	11	rs4754701	100817874	*ARHGAP42*	G/T	0.4978	0.9338	-0.0741	0.0106	2.53E-12	32.250465	0.000287
	12	rs12827990	20018992	*LINC02398*	C/T	0.397	0.466	0.0742	0.0108	7.53E-12	37.116047	0.000332
	12	rs79038319	93125200	*AC138123.1*	C/T	0.3173	0.229	-0.0766	0.0116	4.35E-11	45.556921	0.000409
	20	rs6108787	10986566	*AL050403.2*	T/G	0.552	0.8187	-0.0696	0.0106	5.55E-11	29.161503	0.000259
	3	rs74516251	169112989	*MECOM*	G/A	0.1535	0.1962	-0.0962	0.0148	7.11E-11	29.161503	0.000259
	5	rs1177764	32829869	*NPR3*	C/G	0.6726	0.2872	-0.0696	0.0113	7.18E-10	34.276538	0.000306

	3	rs56194506	169674646	*MECOM*	C/T	0.1106	0.9663	0.1007	0.0167	1.53E-09	23.506526	0.000207
	4	rs1879056	110414448	*ENPEP*	C/T	0.5299	0.6216	0.0592	0.0106	1.60E-08	28.107410	0.000249
	2	rs1405490	61332945	*USP34*	A/G	0.6455	0.2375	-0.062	0.0111	2.07E-08	32.835455	0.000292
	11	rs491205	9746012	*SWAP70*	G/A	0.4056	0.3045	0.0602	0.0107	2.31E-08	24.501659	0.000216
	2	rs77826516	163590845	*FIGN*	C/T	0.551	0.3005	-0.0586	0.0106	3.17E-08	17.495132	0.000151
	8	rs17054793	26038894	*EBF2*	C/T	0.3881	0.6159	0.0602	0.011	4.38E-08	33.367847	0.000297
	3	rs17040476	14876019	*FGD5*	T/C	0.2172	0.7105	0.0705	0.0129	4.84E-08	27.568861	0.000250
Microalbuminuria												
	11	rs11031784	32439029	WT1-AS	T/C	0.7375	0.6024	-0.0952	0.0164	6.22E-09	34.406217	0.000308
	7	rs1971035	29759710	DPY19L2P3	G/A	0.2881	0.5378	-0.0895	0.0159	1.88E-08	31.049404	0.000280

The abbreviations were the same to Supplementary Table S3.

After adjustment for sex, age, BMI, and current smoking status (SABS), GWAS analysis identified the total 20, 30 and 2 candidate SNPs associated with cardiometabolic traits, including DM, hypertension and microalbuminuria, respectively. According to the exclusion criteria, after adjusting for each trait, 4 DM-determining and 8 hypertension-determining SNPs with *P*-values below 0.01 were excluded. *P* value was calculated by linear regression. F-statistic >10 was used to check the strength of association between genetic instrumental variable and each these traits (exposure). R^2 indicated the proportion of variance in the exposure phenotype that is accounted for by the SNPs.

Supplementary Table S4. The lead variants in genome-wide association studies for metabolic liver disease and their association with each trait and remnant cholesterol (RC) level without (column of RC level) or with (column of Trait_adj_RC level) adjustment of each trait.

Trait	CHR	Lead SNPs	BP	A1	Genes	Trait			RC level			Trait_adj_RC level		
						beta	SE	*P*	beta	SE	*P*	beta	SE	P^{a}
NAFLD														
	22	rs16991158	43931299	A	*PNPLA3*	0.3108	0.0265	1.10E-31	-0.0029	0.0024	0.2318	-0.0095	0.0024	7.30E-05
	19	rs58542926	19268740	T	*AC138430.1, TM6SF2*	0.2915	0.0507	9.22E-09	-0.0268	0.0047	1.62E-08	-0.0330	0.0046	8.95E-13
	2	rs6547692	27512105	G	*GCKR*	0.1307	0.0258	4.21E-07	0.0287	0.0024	2.66E-33	0.0260	0.0023	5.38E-29
MAFLD														
	22	rs16991158	43931299	A	*PNPLA3*	0.3121	0.0262	1.15E-32	-0.0014	0.0022	0.5379	-0.0082	0.0021	1.07E-04
MASLD														
	22	rs11090617	43930820	T	*PNPLA3*	0.3161	0.0276	2.64E-30	-0.0031	0.0024	0.2109	-0.0100	0.0024	2.50E-05
	19	rs58542926	19268740	T	*AC138430.1, TM6SF2*	0.2923	0.0526	2.75E-08	-0.0268	0.0047	1.62E-08	-0.0334	0.0046	4.01E-13

Shortened forms: NAFLD: nonalcoholic fatty liver disease; MAFLD: metabolic dysfunction–associated fatty liver disease; and MASLD: metabolic dysfunction–associated steatotic liver disease; the other abbreviations were the same to Supplementary Table S3

GWAS analysis detected the lead SNPs associated with each metabolic disease trait (NAFLD, MAFLD, and MASLD) and RC) level without (column of RC level) or with (column of Trait_adj_RC level) adjustment of each trait, after adjustment for sex, age, BMI, and current smoking status. P^{a} value of <0.01 suggested that association between metabolic liver disease determining-SNPs and RC levels may be independent to the presence of metabolic liver disease.

Supplementary Table S5. The linkage disequilibrium (LD) test between remnant cholesterol level determining lead variants within 1 Mb of the chromosomal region.

Chr.	SNP	BP	Gene	LD (r^2)
11	rs6486122	13,339,977	*ARNTL*	0.008
11	rs7936331	13,812,845	*LINC02548*	
12	rs10744776	109,255,001	*ACACB*	0.004
12	rs75584653	110,021,680	*ANKRD13A*	

Supplementary Figure S3. Summary results for a genome-wide association study of the remnant cholesterol (RC) levels with FUMA.

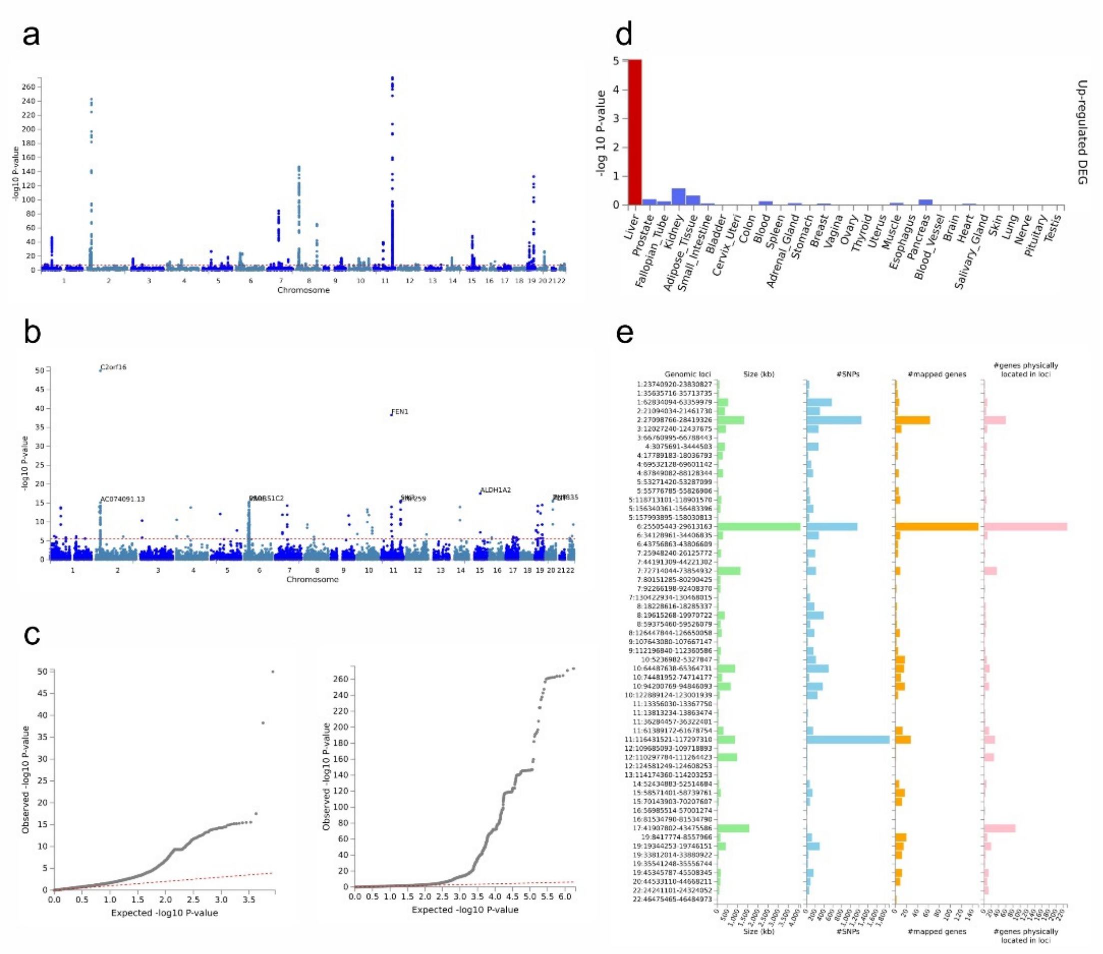

(A) Manhattan plot of the input data of the GWAS of RC levels. (B) With these GWAS summary statistics, gene-based analysis was conducted using MAGMA. (C) The gray dots in Q-Q plot indicate the observed variants, and the red line indicates the expected values under the null hypothesis, indicating no association. (D) Tissue-specific analysis revealing the upregulation of differentially expressed genes in the liver relative to 29 other tissue types (shown in red). (E) The vertical axis represents the format of genomic risk loci as “chromosome: start position–end position.” From left to right, the histograms illustrate the size and number of candidate variants, the number of variants mapped to genes through eQTL mapping, and the number of genes located within the genomic locus.

Supplementary Figure S4. Overview of prioritized genes from a genome-wide association study of the remnant cholesterol (RC) levels with FUMA.

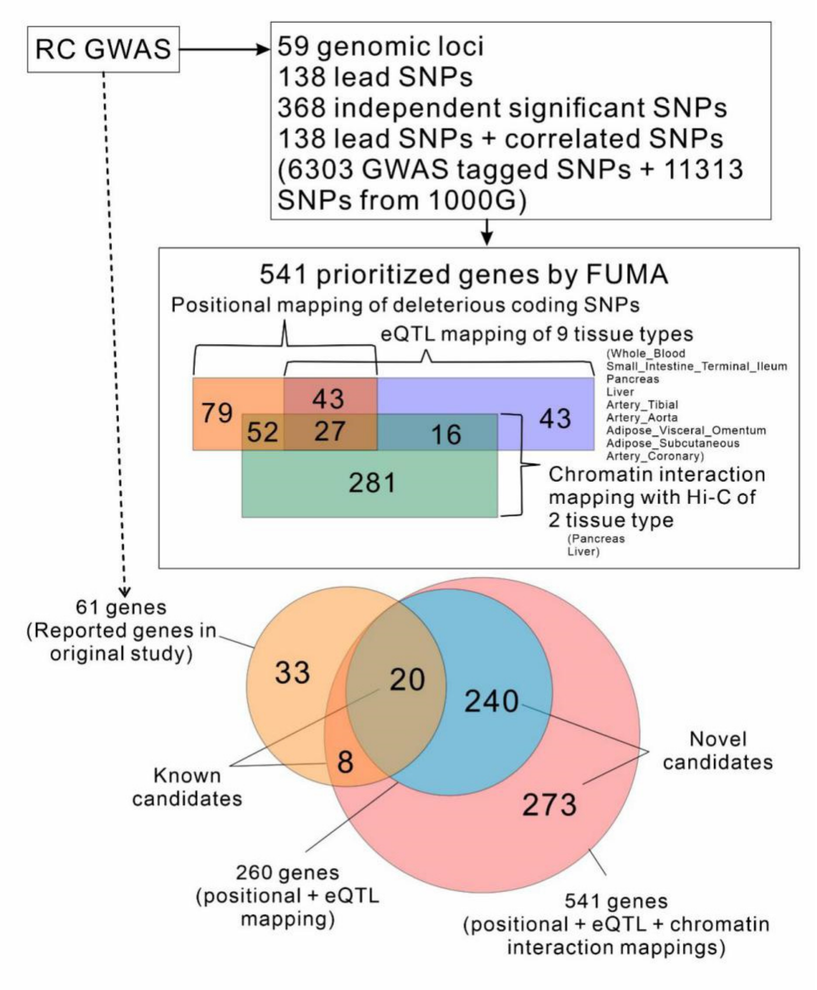

SNP2GENE revealed various annotations in the form of boxes, including 368 independent significant variants and 138 lead variants in LD with independent lead variants. A total of 541 prioritized genes were categorized on the basis of three functional gene mapping approaches: (1) Positional mapping identified genes with deleterious coding variants (201 genes, colored orange); (2) eQTL mapping inferred the association between gene expression and variants on chromosomal regions (129 genes, colored purple); and (3) Chromatin interaction mapping investigated interactions between genes and variants within regulatory regions of chromatin (376 genes, colored green). In addition, the prioritized genes were divided into genes reported in previous studies (orange circle) and novel candidate genes (pink and blue segments) with FUMA.

Supplementary Figure S5. The chromatin interaction and eQTLs of genomic risk loci for remnant cholesterol (RC) levels on chromosomes.

The results depicting the chromatin regions overlapping with RC levels' risk loci through 3D chromatin interaction and eQTLs mappings were presented in FUMA circos plots. In the outermost layer, the rsID and Manhattan plots revealed the top variants in each risk locus and all variants with a significance level of $p < 0.05$ in genomic risk loci, respectively. Within the Manhattan plot, candidate variants were color-coded based on their maximum r^2 correlation with one of the independently significant variants (red: $r^2 > 0.8$, orange: $r^2 > 0.6$, green: $r^2 > 0.4$, and blue: $r^2 > 0.2$). Grey dots represent other variants with $r^2 \leq 0.2$, indicating no linkage disequilibrium. The genomic risk loci are highlighted in navy blue in the second layer, corresponding to chromosome coordinates. In the inner layer, genes are mapped through either chromatin interaction or eQTLs. Genes mapped solely by chromatin interaction or eQTLs are labeled orange or green, respectively. Genes mapped by both mechanisms are colored red. The links representing chromatin interaction and eQTLs are colored orange and green, respectively.

Chromosome 1

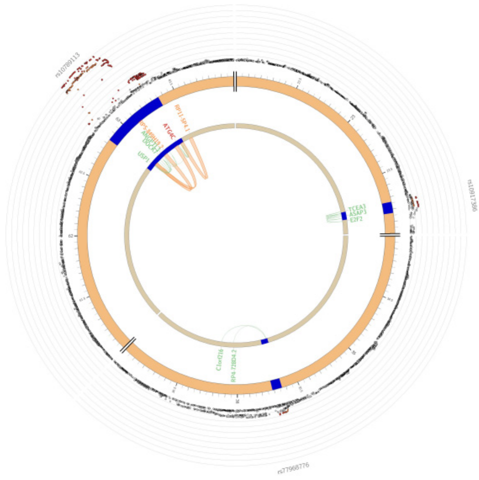

Chromosome 2

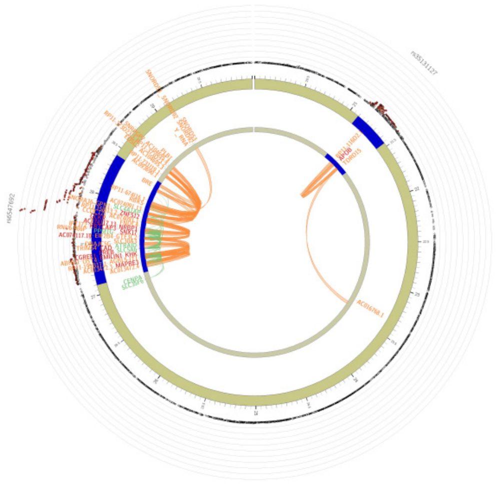

Chromosome 3

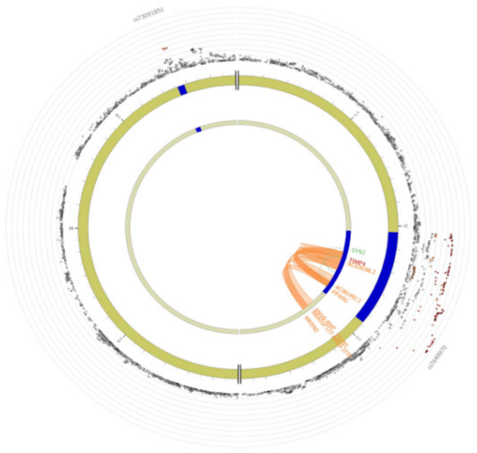

Chromosome 4

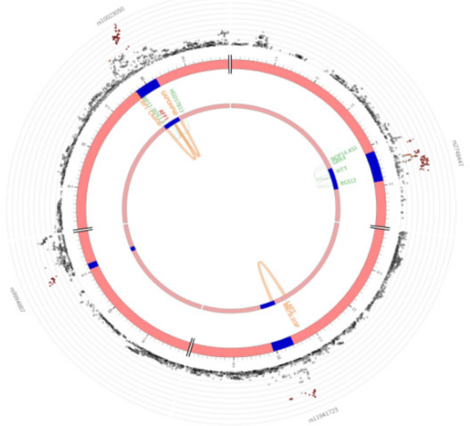

Chromosome 5

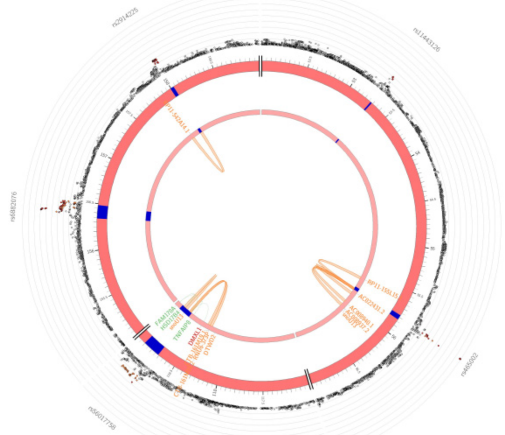

Chromosome 6

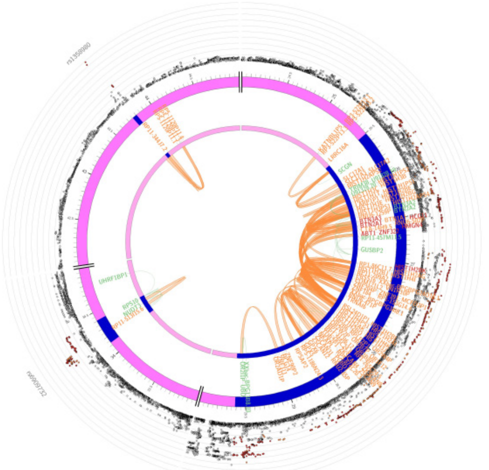

Chromosome 7

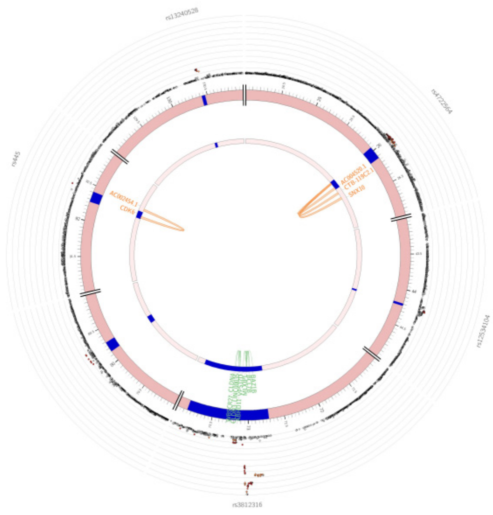

Chromosome 8

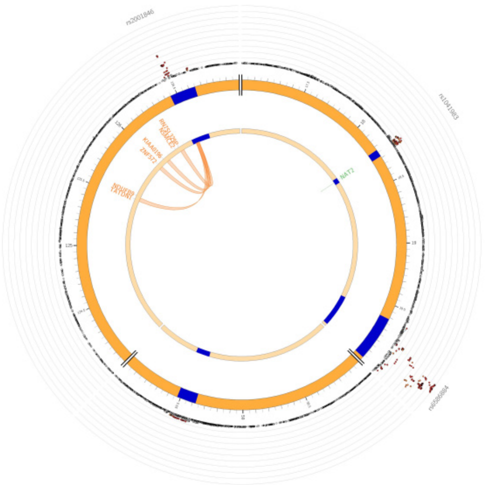

Chromosome 9

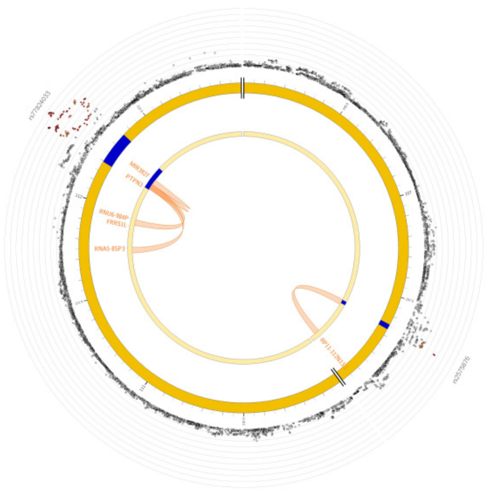

Chromosome 10

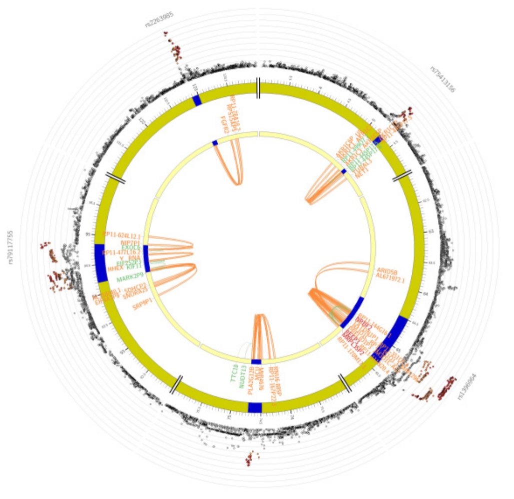

Chromosome 11

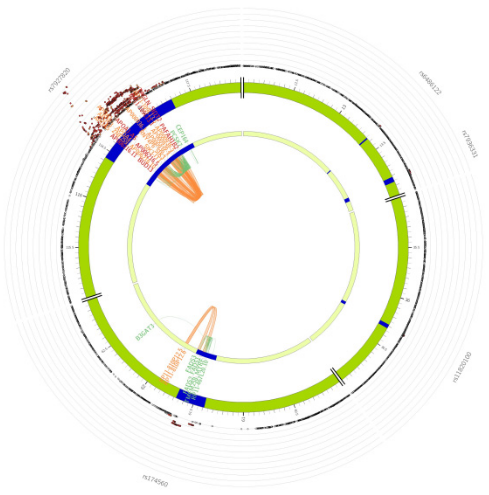

Chromosome 12

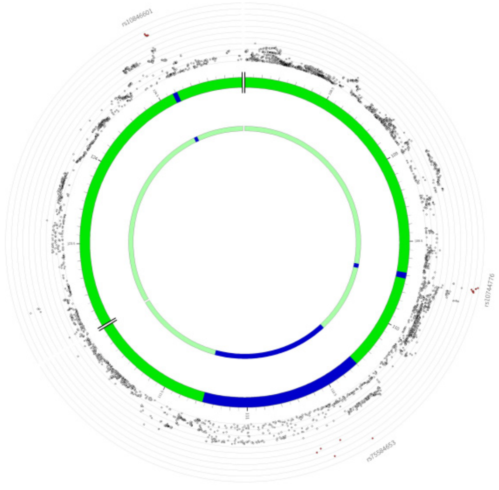

Chromosome 13

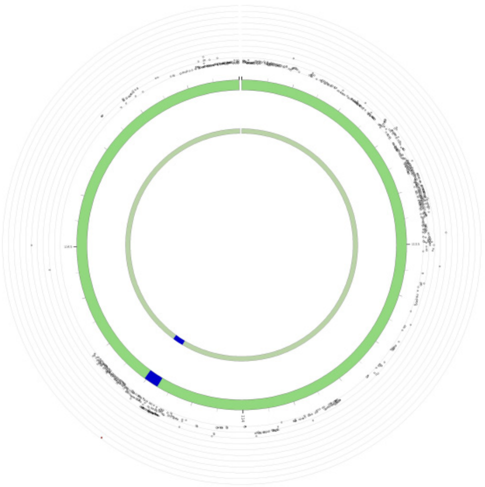

Chromosome 14

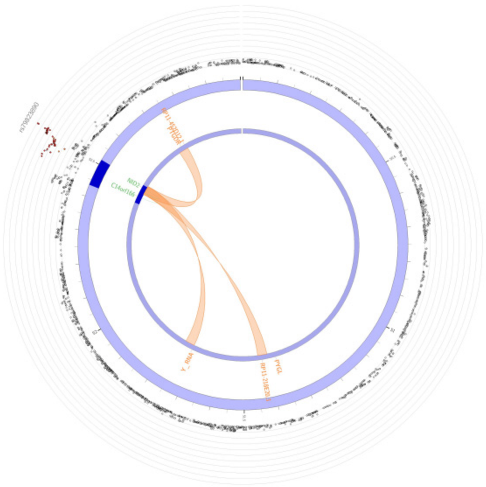

Chromosome 15

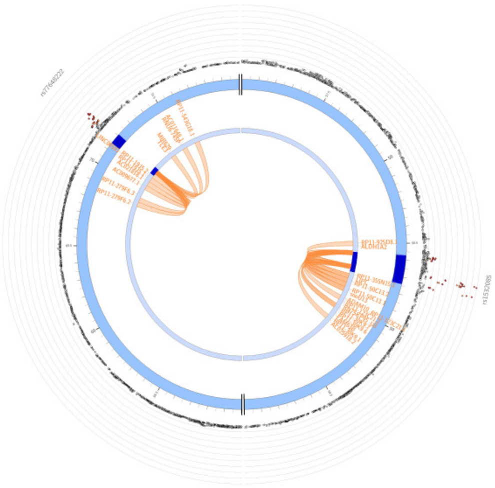

Chromosome 16

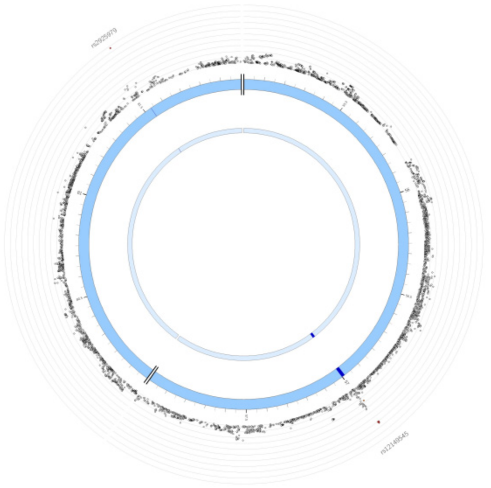

Chromosome 17

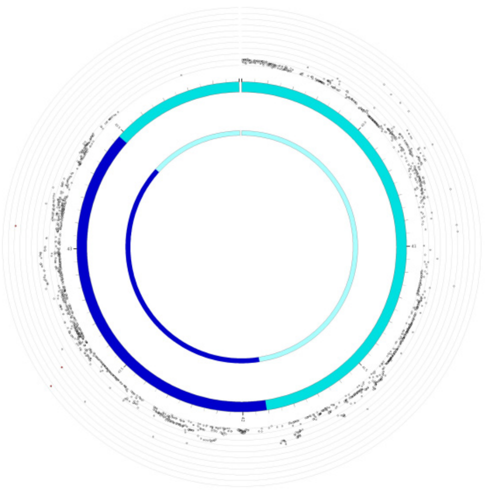

Chromosome 19

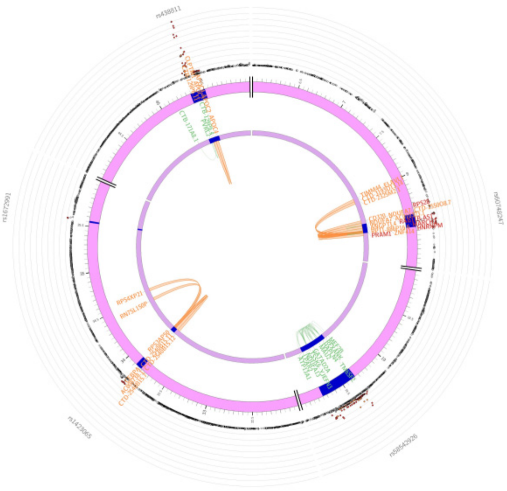

Chromosome 20

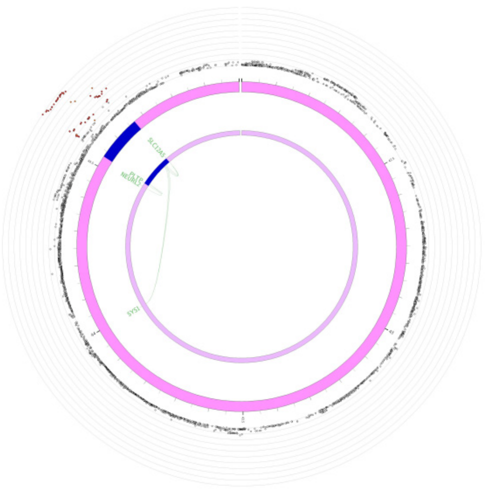

Chromosome 22

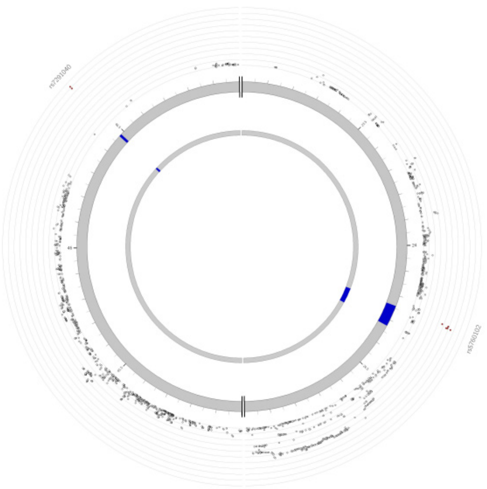

Supplementary Figure S6. Estimates of the causal links between RC-WGRSs and cardiometabolic traits by standard MR methods.

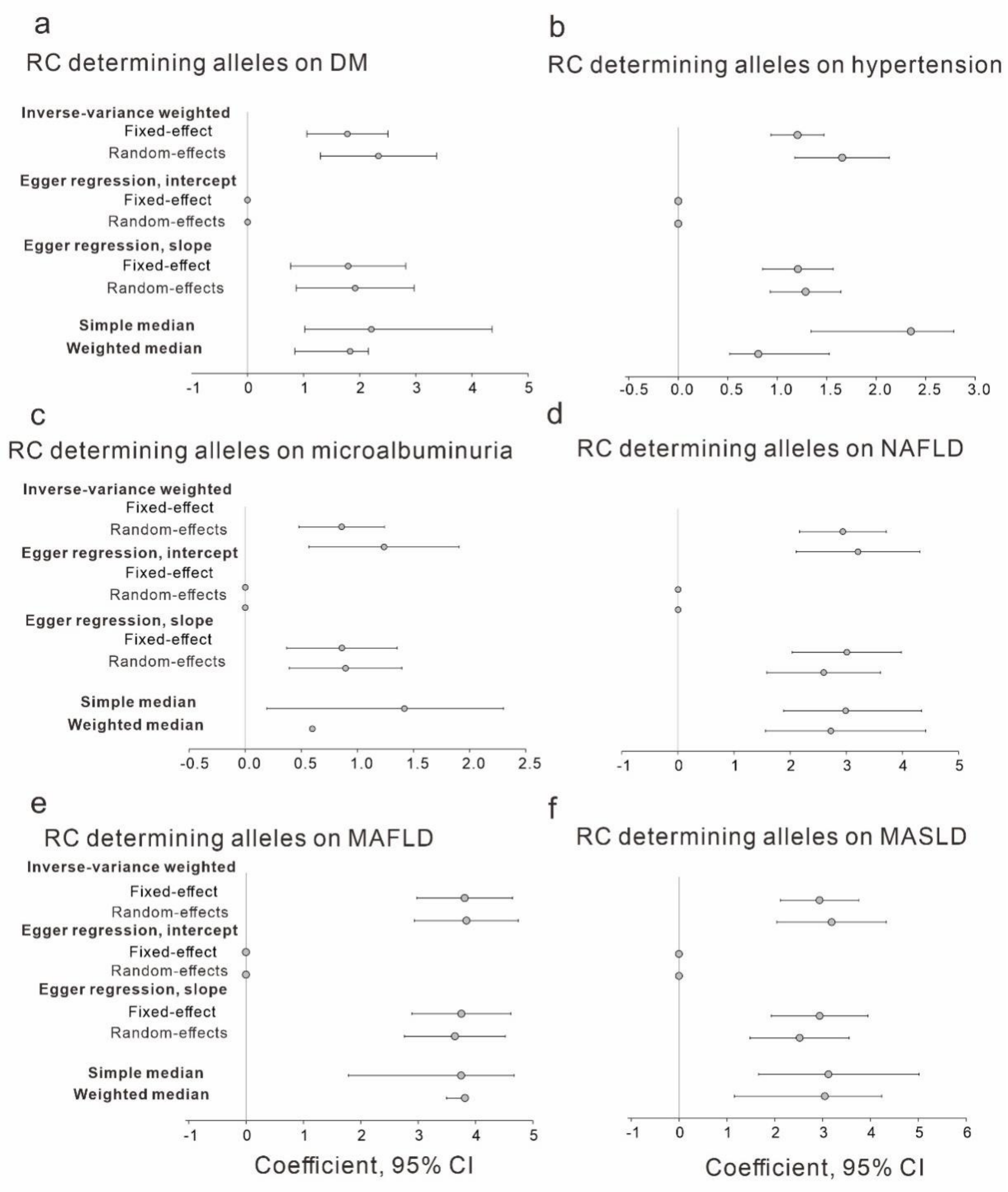

Various MR statistic method, including IVW fixed-effects, IVW random-effects, MR–Egger regression (intercept and slope), simple median, and weighted median, were used to estimate the predicted causal effects of RC-WGRSs on cardiometabolic traits. Abbreviations are the same as those in Supplementary Table S1 and Figure 1.

Supplementary Figure S7. Hypothetical funnel plots of the causal effect estimate between RC-WGRSs and cardiometabolic traits.

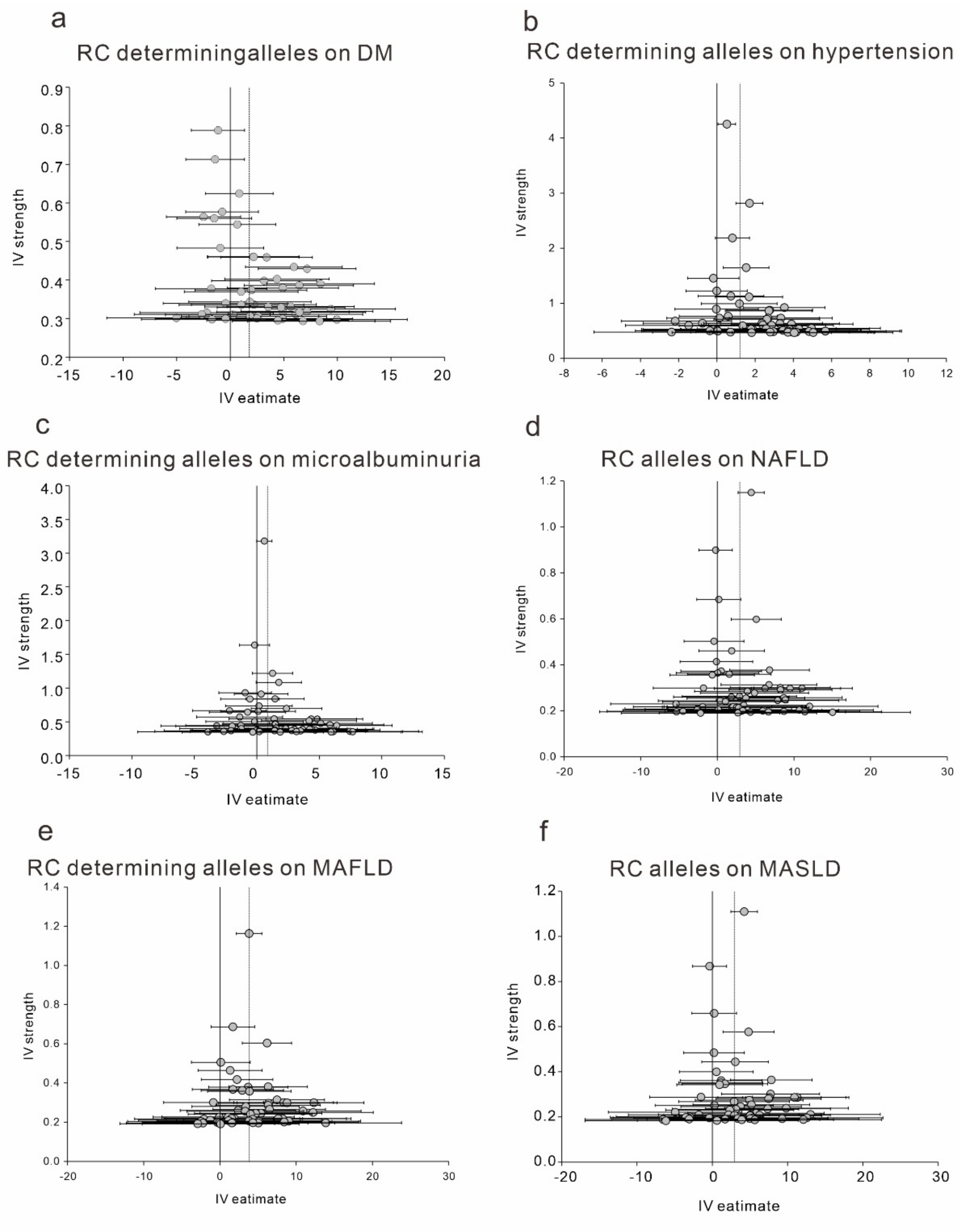

The funnel plots depict the strength of the instrumental variable (IV) against IV estimates for each genetic variant individually in the standard MR analysis for RC on cardiometabolic traits. The x-axis represents the 95% confidence intervals for the IV estimates. Solid vertical lines are positioned at zero, while dashed vertical lines correspond to the IVW with fixed-effect estimates,

revealing asymmetry as a potential indicator of directional pleiotropy. Instrumental variable strength: ($\frac{\widehat{\beta x}\ |G}{SE\ (\widehat{\beta}Y|G)}$) and instrumental variable estimates: ($\frac{\widehat{\beta Y}\ |G}{\widehat{\beta X}\ |G}$).

Supplementary Figure S8. Scatter plots of putative causal relationships between RC-determining alleles on cardiometabolic traits.

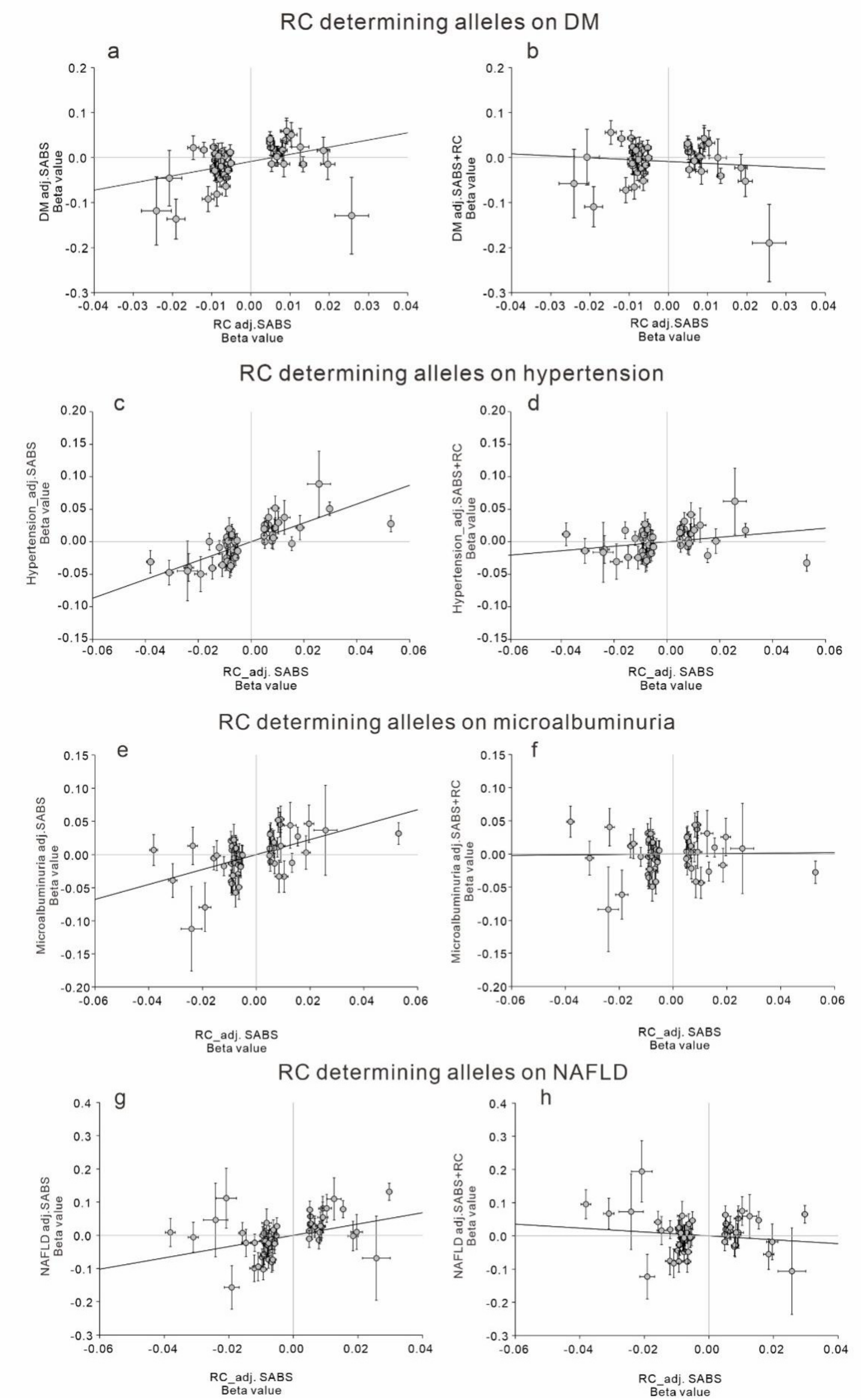

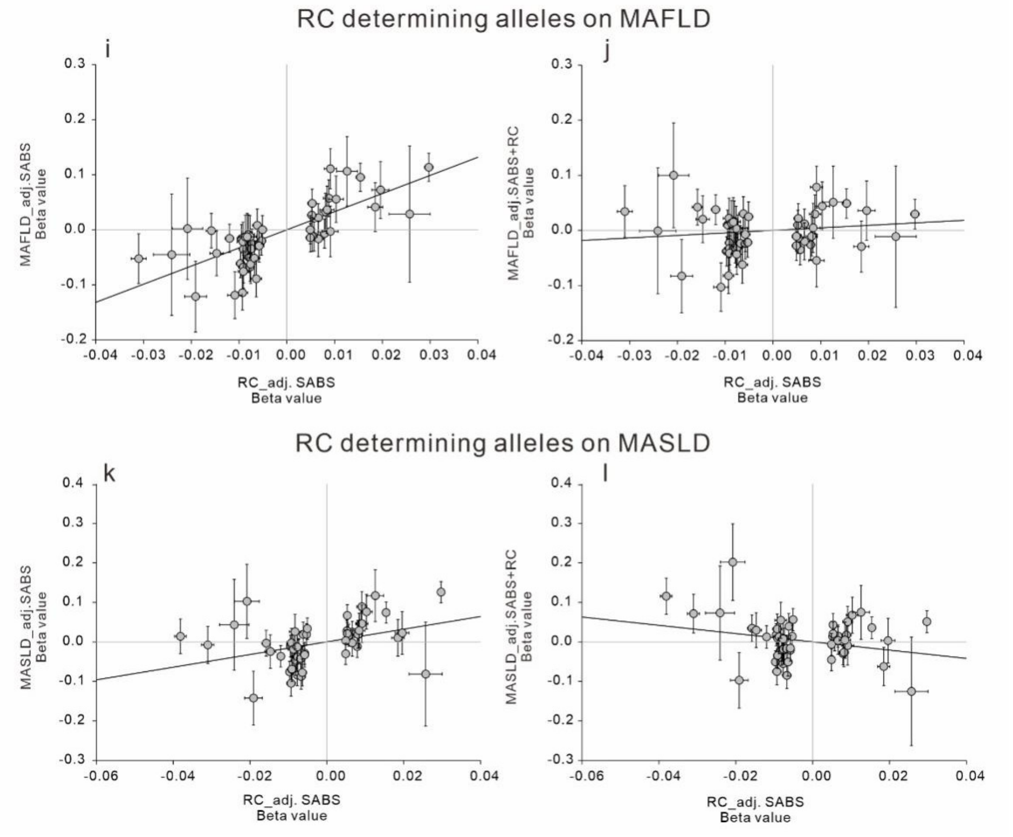

After adjusting for sex, age, BMI, and smoking (SABS), we estimated the effect sizes of alleles determining RC on RC levels (x-axis) and intermediate cardiometabolic traits (Y-axis, including DM, hypertension, microalbuminuria, NAFLD, MAFLD and MASLD). These estimates were presented in scatter plots on the left panel and right panel, either without or with adjustments for RC, respectively. The lines indicated the variability represented by one standard error (s.e.).

Supplementary Figure S9. Estimates of the causal links between cardiometabolic traits-WGRSs and remnant cholesterol (RC) levels in standard MR sensitivity analysis.

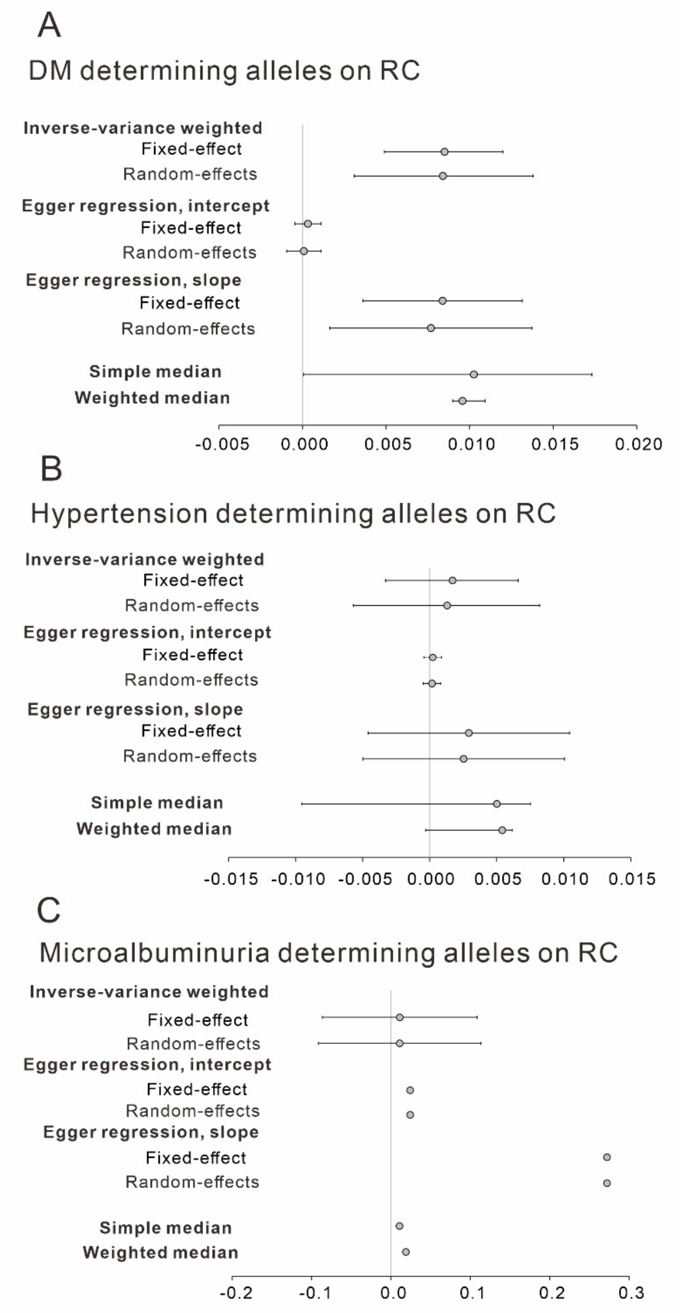

The multiple MR statistic methods, including IVW fixed-effects, IVW random-effects, MR–Egger regression (intercept and slope), simple median, and weighted median, were used to estimate the causal effects of DM, hypertension or microalbuminuria-WGRSs on RC levels.

Supplementary Figure S10. Hypothetical funnel plots of the causal effect estimate between cardiometabolic traits-WGRSs and remnant cholesterol (RC) levels.

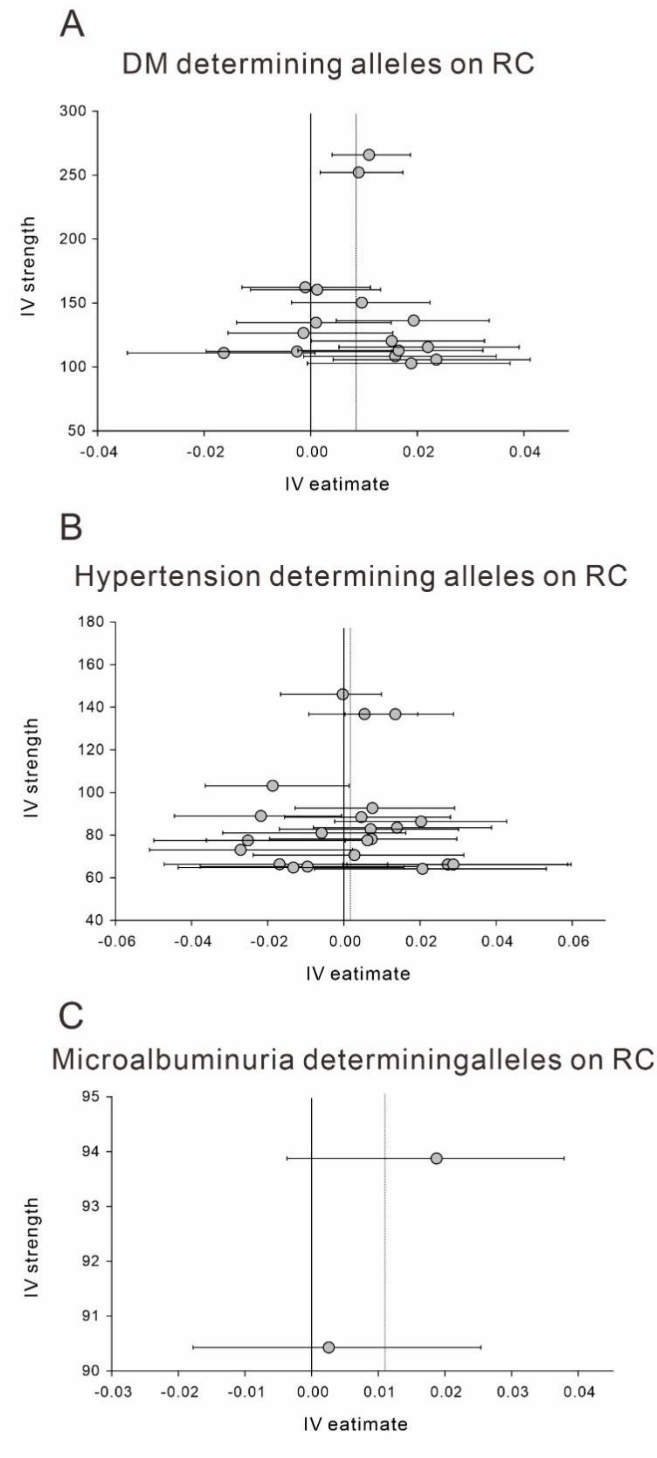

The funnel plots depict the strength of the instrumental variable (IV) against IV estimates for each genetic variant individually in the standard MR analysis for DM, hypertension and microalbuminuria on RC levels. The x-axis represents the 95% confidence intervals for the IV

estimates. Solid vertical lines are positioned at zero, while dashed vertical lines correspond to the IVW with fixed-effect estimates, revealing asymmetry as a potential indicator of directional pleiotropy.

Supplementary Figure S11. Scatter plots of putative causal relationships between RC-determining alleles on cardiometabolic traits.

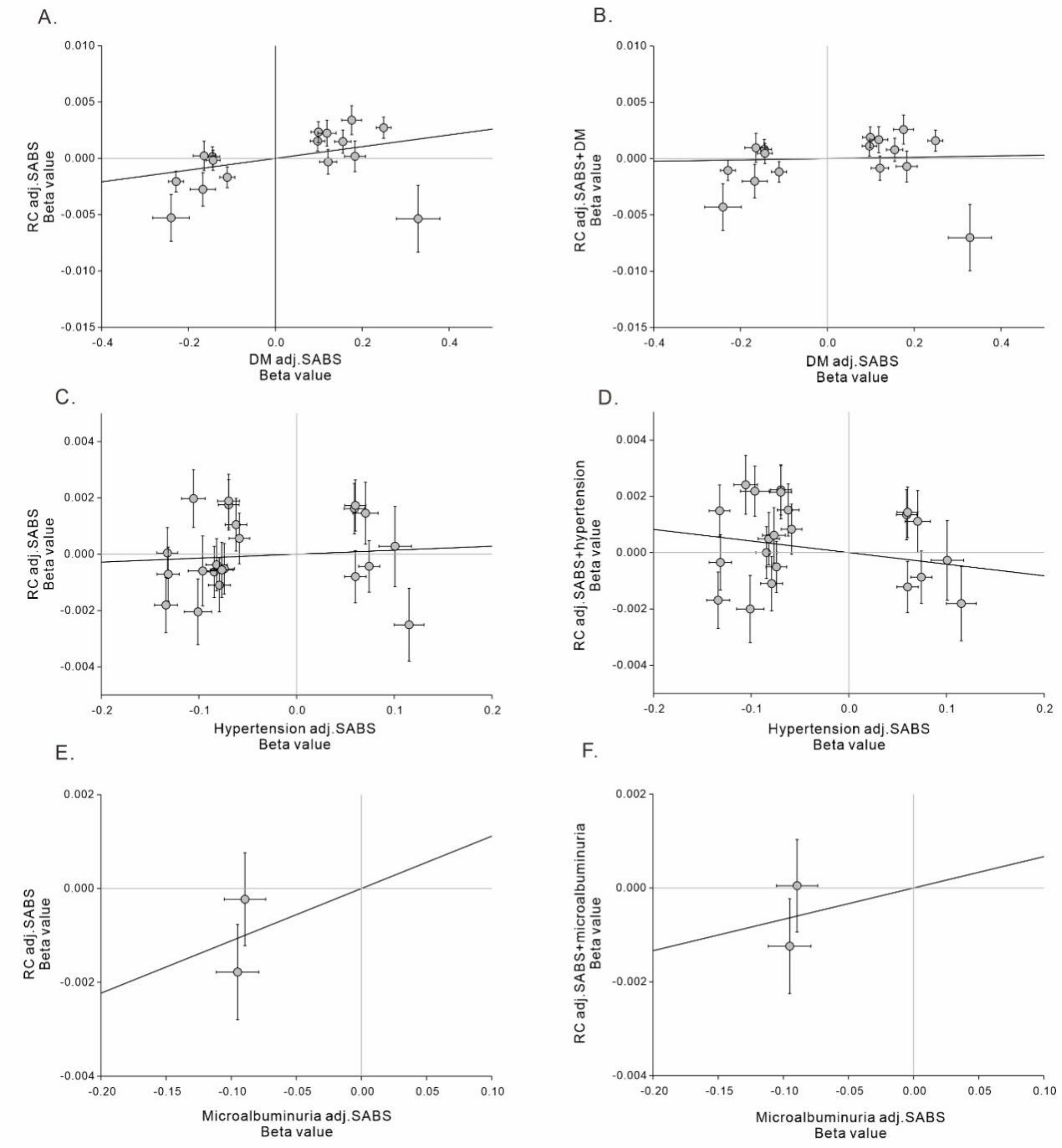

After adjusting for sex, age, BMI, and smoking (SABS), we estimated the effect sizes of cardiometabolic traits (i.e. DM, hypertension, and microalbuminuria) determining alleles (x-axis) and RC levels (Y-axis). These estimates were presented in scatter plots on the left panels and right panels, either without or with adjustments for each these traits, respectively. The lines indicated the variability represented by one standard error (s.e.).

Supplementary Table S13. Cochran's Q and Rücker's Q tests for heterogeneity

Exposure [E]	Outcome [O]	Instrumental variables	Analysis Method*	Coefficient*	Standard error	95% confident interval	*P* value	Cochran's Q $_{IVW}$ / Rücker's Q $_{MR-Egger}$**	*P1* value***
RC	DM	RC -determining alleles	IVW method	1.7806	0.3587	1.0585 - 2.5027	6.92×10^{-7}	88.5090	0.0002
			Egger regression, slope	1.7945	0.5095	0.7684 - 2.8206	0.0010	88.4603	0.0002
RC	Hypertension	RC -determining alleles	IVW method	1.2059	0.1332	0.9387 - 1.4731	$< 10^{-307}$	90.7200	0.001
			Egger regression, slope	1.2104	0.1771	0.8548 - 1.5660	9.96×10^{-9}	108,7200	< 0.00001
RC	Microalbuminuria	RC -determining alleles	IVW method	0.8597	0.1897	0.4790 - 1.2405	5.86×10^{-6}	84.9500	0.0027
			Egger regression, slope	0.8625	0.2445	0.3716 - 1.3533	0.0009	84.6881	0.0028
RC	NAFLD	RC -determining alleles	IVW method	2.9390	0.3866	2.1643 - 3.7137	2.91×10^{-14}	83.4900	0.0080
			Egger regression, slope	3.010827	0.484426	2.039611 - 3.982043	7.69×10^{-8}	82.3681	0.0099
RC	MAFLD	RC -determining alleles	IVW method	3.8098	0.4153	2.9761 - 4.6436	$< 10^{-307}$	53.77	0.3690
			Egger regression, slope	3.7523	0.429780	2.889037 - 4.615513	1.26×10^{-11}	70.05	0.0700
RC	MASLD	RC -determining alleles	IVW method	2.9338	0.4069	2.1174 - 3.7502	5.56×10^{-13}	80.3862	0.0090
			Egger regression, slope	2.9360	0.5026	1.9270 - 3.9450	3.63×10^{-7}	75.7260	0.0175
DM	RC	DM-determining alleles	IVW method	0.0085	0.0017	0.0049 - 0.0120	3.64E-07	26.26	0.035
			Egger regression, slope	0.008385	0.002223	0.003617 - 0.013152	0.002061	26.81	0.030332
Hypertension	RC	Hypertension-determining alleles	IVW method	0.0017	0.0024	-0.0033 to 0.0066	0.4837	35.90	0.022
			Egger regression, slope	0.002914	0.003598	-0.004591 to 0.010418	0.427531	25.00856	0.246818

Microalbiminuria	RC	Microalbiminuria-determining alleles	IVW method	0.0110	0.0077	-0.0865 to 0.1084	0.1533	1.11	0.293
			Egger regression, slope	0.272189	0	0.272189 - 0.272189	--	1.54E-07	0.999691

RC: remnant cholesterol; IVW: Inverse-variance weighted

*All with fixed effect

**For IVW method, Cochran's Q test was performed and for Egger regression method, we used Rücker’s Q test

****P1* value: *P* value for the Cochran's Q or Rücker’s Q tests
