# Supplementary material for: Causal Associations Between Remnant Cholesterol Levels and Atherosclerosis-Related Cardiometabolic Risk Factors: A Bidirectional Mendelian Randomization Analysis
Source: Genes (Basel). 2025 Jan 26;16(2):157. doi: 10.3390/genes16020157 (PMC11855473; doi:10.3390/genes16020157)
Supplement: Supplementary file 1 [file genes-16-00157-s001.zip › Supplementary_Methods.pdf]

Causal associations between remnant cholesterol levels and atherosclerosis-related cardiometabolic risk factors: A bidirectional Mendelian randomization analysis

Supplementary Method S1

Definitions of current smoking, alcohol drinking, exercise, hypertension, chronic kidney disease, non-alcoholic fatty liver disease (NAFLD), metabolic dysfunction-associated fatty liver disease (MAFLD), metabolic associated steatotic liver disease (MASLD), carotid intimal-medial thickness (CIMT), Carotid plaque, DM, microalbuminuria, metabolic syndrome

Current smoking was defined as regular cigarette smoking reported by the participants at the time of case receipt and lasting more than 6 months. "Alcohol consumption" was defined as regular consumption of alcohol, with a threshold of consuming 150 ml per week consistently for more than 6 months. **Exercise** was defined based on the frequency of physical training, with participants engaging mostly 3-4 times per week, and each session lasting mostly 30 to 60 minutes. The definition of **Hypertension** was characterized by an average systolic blood pressure (BP) of ≥ 130 mmHg and/or diastolic BP of ≥ 85 mmHg over at least three consecutive measurements under standard measurement methods, or a self-stated history by the participant. The definition of **Chronic kidney disease** indicated a decline in renal function, an estimated glomerular filtration rate (eGFR) of < 60 mL/min/1.73 m^2 of body surface area. According to the Consensus Statement outlined by the American Society of Echocardiography, **carotid intimal-medial thickness** (CIMT) values equal or greater than the 75th percentile of the distribution of a healthy population entail a risk higher than that estimated by the Framingham risk score (Stein et al., 2008) and thus, in this study, abnormal CIMT was defined as the average distance of 0.7 mm or above between the lumen–intima interface and the media–adventitia interface of the right and left common carotid arteries. **Carotid plaque**, a fatty deposit, was a sign of atherosclerosis and served as a marker of atherosclerotic severity (Kabłak-Ziembicka et al., 2021). In both with and without the use of ultrasound contrast agents, visible carotid plaques were defined as localized thickening invading the lumen of any segment of the carotid artery, considered to be caused by atherosclerosis. The definition of **diabetes mellitus** (DM)

included fasting blood glucose level of ≥7.0 mmol/L, a glycosylated hemoglobin (HbA1c) value of ≥6.5%, or a self-stated history of DM. To evaluate albuminuria, spot urine albumin levels were exclusively used because of the absence of urine creatinine levels, with **Microalbuminuria** being defined as a urine albumin level of ≥30 mg/L. Metabolic liver disease was defined according to three different evolutional terms, including **non-alcoholic fatty liver disease** (NAFLD), **metabolic dysfunction-associated fatty liver disease** (MAFLD), and **metabolic dysfunction-associated steatotic liver disease** (MASLD). The definitions of NAFLD, MAFLD, and MASLD were shown in Supplementary Method Figure S1 below (Rinella et al., 2023; Bilson et al., 2024).

Metabolic syndrome characteristics were determined based on the modified criteria from the third report of the National Cholesterol Education Program's Adult Treatment Panel III, given the absence of medication histories (Grundy et al., 2005). Participants are defined as having metabolic syndrome if they exhibit three or more of the following characteristics: (1) BP ≥130/85 mmHg or a documented history of hypertension; (2) triglyceride level ≥1.70 mmol/L; (3) high-density lipoprotein cholesterol level <1.04 mmol/L for men or <1.30 mmol/L for women; (4) fasting plasma glucose ≥5.6 mmol/L or a documents history of DM; and (5) WC >90 cm for men or >80 cm for women.

Participants who developed individual cardiometabolic risk factors such as DM, hypertension, or CKD during the follow-up period, without having those risk factors during the initial survey, were considered to have new onset of these conditions.

Supplementary Method Figure S1. Definitions of NAFLD, MAFLD, and MASLD.

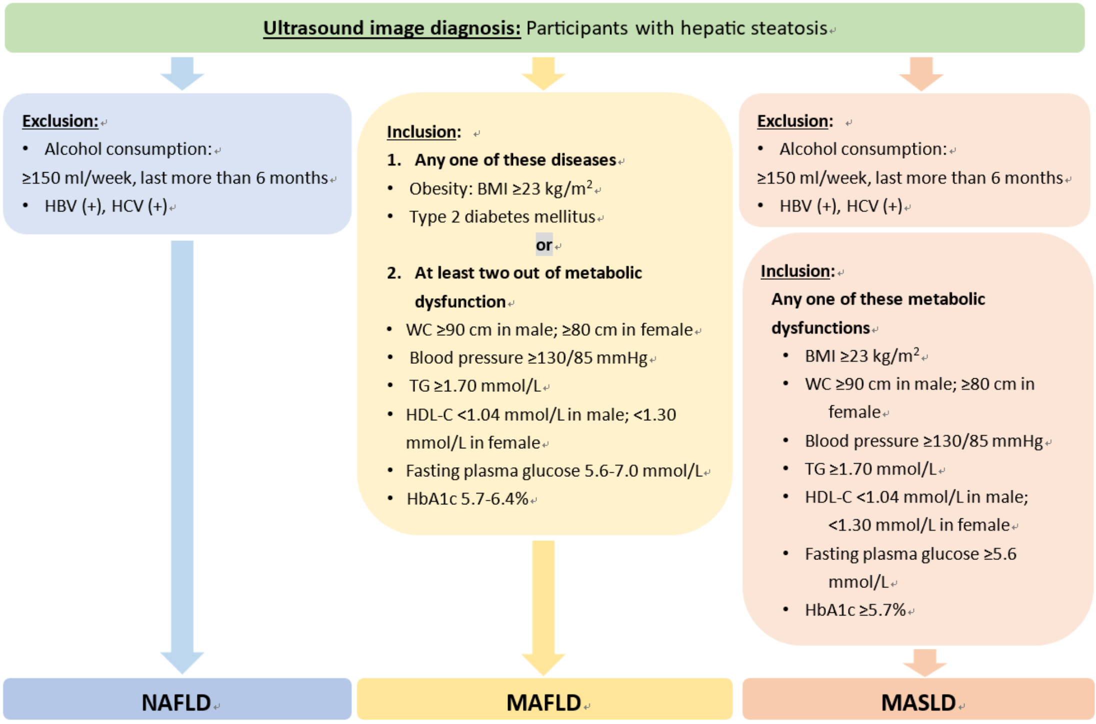

Abbreviation: HBV: hepatic B virus; HCV: hepatic C virus; BMI: body mass index; WC: waist circumference; TG: triglycerides; HDL-C: high-density lipoprotein-cholesterol; HbA1c: glycosylated hemoglobin; hs-CRP: high sensitivity C-reactive protein; HOMA-IR, homeostatic model for assessment of insulin resistance; NAFLD: non-alcoholic fatty liver disease; MAFLD: metabolic dysfunction-associated fatty liver disease; MASLD: metabolic dysfunction-associated steatotic liver disease.

Reference:

1. Stein J.H., Korcarz C.E., Hurst R.T., et al. Use of carotid ultrasound to identify subclinical vascular disease and evaluate cardiovascular disease risk: a consensus statement from the American Society of Echocardiography Carotid Intima-Media Thickness Task Force. Endorsed by the Society for Vascular Medicine. J. Am. Soc. Echocardiogr. 2008;21:93–111.
2. Kabłak-Ziembicka A, Przewłocki T. Clinical Significance of Carotid Intima-Media Complex and Carotid Plaque Assessment by Ultrasound for the Prediction of Adverse Cardiovascular Events in Primary and Secondary Care Patients. J Clin Med. 2021; 10:4628.

3. Rinella ME, Lazarus JV, Ratziu V, Francque SM, Sanyal AJ, Kanwal et al. A multisociety Delphi consensus statement on new fatty liver disease nomenclatur e. Hepatology. 2023 Dec 1;78(6):1966-1986.
4. Bilson J, Mantovani A, Byrne CD, Targher G. Steatotic liver disease, MASLD and risk of chronic kidney disease. Diabetes Metab. 2024 Jan;50(1):101506.
5. Grundy SM, Cleeman JI, Daniels SR, Donato KA, Eckel RH, Franklin BA, et al. Diagnosis and management of the metabolic syndrome: an American Heart Association/National Heart, Lung, and Blood Institute Scientific Statement. Circulation. 2005; 11: 2735–52.

Supplementary Method S2

Measurements of LDL-C

LDL-C was measured as follows: If the TG level was ≤3.96 mmol/L, the level of LDL-C was determined by subtracting the sum of HDL-C and one-fifth of TG levels from TC. If the TG level was >3.96 mmol/L, direct measurements of LDL-C levels were conducted using colorimetric assays.

Supplementary Method S3

Genomic DNA extraction, genotyping, and GWAS analysis

Genomic DNA was extracted from the whole blood of participants. The genotype data derived from Axiom Genome-Wide TWBv1.0 and TWBv2.0 Arrays, with a total of 27,685 and 103,316 participants, respectively, were used to perform GWAS after imputation (Yeh, et al., 2022 and Chou, et al., 2022). Participants (n = 1,459) whose data were obtained from both arrays were not duplicated in our analysis. The bcftools software was used to assess genotype concordance between TWBv1.0 and TWBv2.0, revealing a consistency rate of 99.86%. Moreover, whole-genome sequencing was used to further confirm the genotyping data of both arrays, showing a consistency rate of 99.92% and 99.80% for TWBv1.0 and TWBv2.0, respectively. Only variants displaying complete concordance between the two arrays were retained for imputation. Subsequently, the software packages SHAPEIT (version 2; Oxford, UK; https://mathgen.stats.ox.ac.uk/genetics_software/shapeit/shapeit.html, accessed December 2, 2020) and IMPUTE2 (version 2; Oxford, UK; http://mathgen.stats.ox.ac.uk/impute/impute_v2.html, accessed December 2, 2020) were used to perform genome-wide genotype imputation, with the 1000 Genomes Project Phase 3 East Asian Populations serving as the reference panel. After the imputation process, quality control (QC) assessments were conducted. In these assessments, variants with IMPUTE2 imputation quality scores of >0.3 were excluded, and insertion–deletion mutations were removed using VCFtools (version 0.1; https://vcftools.github.io/index.html, accessed December 2, 2020). Additionally, variants included in our sequential examination were required to have a missing rate of <3%, a minor allele frequency of ≥0.01, and a p value for Hardy–Weinberg equilibrium violation of $\geq 10^{-6}$ (Feng et al., 2022). Linear regression methods were employed to analyze GWAS data. A p value of $< 5 \times 10^{-8}$ was considered to indicate genome-wide significance. Beta-coefficient and standard error to predict the effects of genetic variants on RC levels or cardiometabolic and vascular traits. The independence of variants located close to each other (within 1 Mb) on the same chromosome was confirmed through a linkage disequilibrium (LD) test, where the acceptable threshold was set to $r^2 < 0.1$.

References:

1. Yeh KH, Wan HL, Teng MS, Chou HH, Hsu LA, Ko YL. Genetic Variants at the APOE Locus Predict Cardiometabolic Traits and Metabolic Syndrome: A Taiwan Biobank Study. Genes (Basel). 2022;13(8):1366.
2. Chou HH, Hsu LA, Juang JJ, Chiang FT, Teng MS, Wu S, Ko YL. Synergistic Effects of Weighted Genetic Risk Scores and Resistin and sST2 Levels on the Prognostication of Long-Term Outcomes in Patients with Coronary Artery Disease. Int J Mol Sci. 2022;23(8):4292.
3. Feng YA, Chen CY, Chen TT, Kuo PH, Hsu YH, Yang HI, Chen WJ, Su MW, Chu HW, Shen CY, Ge T, Huang H, Lin YF. Taiwan Biobank: A rich biomedical research database of the Taiwanese population. Cell Genom. 2022;2(11):100197.

Supplementary Method S4

Genomic loci definition and functional annotation

As mentioned, FUMA is an online platform (http://fuma.ctglab.nl/) that integrates information from 18 biological databases and analytical tools to facilitate the functional annotation of genes and their associated variants, thus enabling the identification of independent genomic loci (Watanabe, et al., 2017). In this study, independent significant variants were identified using a conditional false discovery rate of <0.01 and a conjunctional false discovery rate of <0.05; their independence was confirmed through a linkage disequilibrium (LD) test, in which the acceptable threshold was set to $r^2 < 0.6$. Lead variants were selected on the basis of their approximate LD with each other ($r^2 < 0.1$). Distinct genomic loci were identified by grouping all lead variants that physically overlapped each other (within LD blocks less than 250 kb apart) and shared a strong genetic correlation ($r^2 \geqq 0.6$) with at least one of the independent significant variants within the locus; these loci were identified using a conditional or conjunctional false discovery rate of <0.01. The genomic region containing all candidate variants was regarded as a single independent genomic locus. To calculate LD information, the 1000 Genomes Project Phase 3 East Asian Populations served as the reference panel (Auton et al., 2015).

Combined annotation-dependent depletion, an annotation algorithm that integrates data from more than 60 genomic features, can be used to score human single-nucleotide variants and short insertions and deletions across a reference genome (Rentzsch et al., 2019); in this study, combined annotation-dependent depletion was used to predict the deleterious effects of the variants on both the structure and function of proteins. Moreover, RegulomeDB (Boyle et al., 2012) was used to predict the transcriptional and regulatory effects of chromatin states at the variant locus. Candidate variants were matched with genes through three distinct approaches in FUMA: mapping genes depending on their physical proximity to variants (positional mapping), associating variants with genes whose expression is affected by allelic variations through expression quantitative trait locus (eQTL) mapping, and linking variants to genes by utilizing information on three-dimensional DNA–DNA interactions to reflect chromatin states (chromatin interaction mapping). A locus was deemed novel if it did

not physically overlap with any previously reported GWAS associations and if its candidate variants were not documented in the GWAS catalog; this essentially served as a novelty check (MacArthur et al., 2017). FUMA was used to conduct a gene-set enrichment analysis (GSEA) for genes that were close to the identified shared loci, as defined by the Gene Ontology (Ashburner et al., 2000). These gene mapping strategies were used to develop curated pathways. For tissue-specific analysis, the expression levels of prioritized genes represent log2-transformed average transcripts per million per tissue type, winsorized at the 50th percentile. These levels were derived from RNA-seq data obtained from Genotype-Tissue Expression (GTEx) that was used to biologically contextualize potential regulatory lead variants (Battle et al., 2017).

References:

1. Watanabe, K., E. Taskesen, A. van Bochoven, and D. Posthuma. 2017. Functional mapping and annotation of genetic associations with FUMA. Nature communications 8: 1826.

2. Auton, A., L. D. Brooks, R. M. Durbin, E. P. Garrison, H. M. Kang, J. O. Korbel, J. L. Marchini, S. McCarthy, G. A. McVean, and G. R. Abecasis. 2015. A global reference for human genetic variation. Nature 526: 68-74.

3. Rentzsch, P., D. Witten, G. M. Cooper, J. Shendure, and M. Kircher. 2019. CADD: predicting the deleteriousness of variants throughout the human genome. Nucleic acids research 47: D886-d894.

4. Boyle, A. P., E. L. Hong, M. Hariharan, Y. Cheng, M. A. Schaub, M. Kasowski, K. J. Karczewski, J. Park, B. C. Hitz, S. Weng, J. M. Cherry, and M. Snyder. 2012. Annotation of functional variation in personal genomes using RegulomeDB. Genome research 22: 1790-1797.

5. MacArthur, J., E. Bowler, M. Cerezo, L. Gil, P. Hall, E. Hastings, H. Junkins, A. McMahon, A. Milano, J. Morales, Z. M. Pendlington, D. Welter, T. Burdett, L. Hindorff, P. Flicek, F. Cunningham, and H. Parkinson. 2017. The new NHGRI-EBI Catalog of published genome-wide association studies (GWAS Catalog). Nucleic acids research 45: D896-d901.

6. Ashburner, M., C. A. Ball, J. A. Blake, D. Botstein, H. Butler, J. M. Cherry, A. P. Davis, K. Dolinski, S. S. Dwight, J. T. Eppig, M. A. Harris, D. P. Hill, L. Issel-Tarver, A. Kasarskis, S. Lewis, J. C. Matese, J. E. Richardson, M. Ringwald, G. M. Rubin, and

G. Sherlock. 2000. Gene ontology: tool for the unification of biology. The Gene Ontology Consortium. Nature genetics 25: 25-29.

7. Battle, A., C. D. Brown, B. E. Engelhardt, and S. B. Montgomery. 2017. Genetic effects on gene expression across human tissues. Nature 550: 204-213.

Supplementary Method S5

Sensitivity analysis for causal inference from standard Mendelian randomization (MR) with multiple genetic variants

To simultaneously investigate the causal relationships between dependent and independent variables, we initially conducted multivariate analysis to assess the potential bias from horizontal pleiotropy and mitigate the influence of measurable confounding factors. **Funnel plots**, a simple method for detecting directional pleiotropy, were used to illustrate the causal precisions of instrumental variables (IVs) on the vertical axis against the IV estimates for each genetic variant on the horizontal axis. The ratios of the IV estimates obtained from individual genetic variants were combined using a formula based on **inverse-variance weighted (IVW)** method (Burgess et al., 2013; Johnson et al., 2012) to derive an overall estimate. The summary of IVW analysis give similar mean and median estimates to the two stage least square (2SLS) that commonly used with individual-level data. The IVW method remains unbiased as the number of single nucleotide variation increases. In IVW model, fitting a random effect was employed to receive the corrected standard error (Bowden et al., 2017). The IVW estimate proved to be an efficient analysis method when all genetic variants were deemed valid IVs. Conversely, a **simple median estimator** offered a reliable estimate of the causal effect when fewer than 50% of the examined genetic variants meet the validity criteria (Bowden et al., 2016). The IVW regression analysis was conducted using Meta-Essentials_1.4, an analytical software available at www.erim.eur.nl/research-support/meta-essentials. When there was considerable variability in individual estimates, relying solely on the simple median estimator proves inadequate. In such cases, the **weighted median emerges** as a more robust method. This method utilizes the median of the weighted ratio, employing standardized weights to ensure that the sum of the weights equals 1. Notably, the weighted median furnishes a consistent estimate provided that at least 50% of the weight stems from valid IVs. SPSS 22 statistical software (SPSS Inc, Chicago, IL) was used to perform both forms of median regression. The **MR-Egger regression** approach, originating from the field of meta-analysis (Egger et al., 1997), served the purpose of evaluating small-study biases and identifying overall directional pleiotropy across distinct genetic variants

when the intercept term is not equal to zero (Bowden et al., 2016). It offers a reliable estimation of the genuine causal effect, maintaining consistency even when all genetic variants fail to meet the instrumental variable (IV) assumption criteria, as described by Bowden et al. (2016). We implemented this methodology utilizing Meta-Essentials_1.4 and SPSS analysis software.

On the basis of the approach outlined by Dai, et al. (2014), **scatter plots** were evaluated the concordance of IV effects. We considered the estimated causal associations to be strong if the independent genetic variants from the various gene regions exhibited concordant associations with the outcomes of interest. Heterogeneity was examined through the visual inspection of the scatter plots and the application of **Cochran's or Rücker's Q tests** on the causal estimate for each genetic variant in relation to the outcome of interest, in comparison to the genetic association of the exposure of interest. The methodologies for these assessments are detailed in the works of Burgess, et al. (2018) and Bowden, et al. (2018).

Reference:

1. Bowden, J., Davey Smith, G., Haycock, P. C., & Burgess, S. 2016. Consistent Estimation in Mendelian Randomization with Some Invalid Instruments Using a Weighted Median Estimator. Genet Epidemiol, 40(4): 304-314.
2. Bowden, J., Del Greco, M. F., Minelli, C., Davey Smith, G., Sheehan, N., & Thompson, J. 2017. A framework for the investigation of pleiotropy in two-sample summary data Mendelian randomization. Stat Med, 36(11): 1783-1802.
3. Burgess, S., Bowden, J., Fall, T., Ingelsson, E., & Thompson, S. G. 2017. Sensitivity Analyses for Robust Causal Inference from Mendelian Randomization Analyses with Multiple Genetic Variants. Epidemiology, 28(1): 30-42.
4. Burgess, S., Butterworth, A., & Thompson, S. G. 2013. Mendelian randomization analysis with multiple genetic variants using summarized data. Genet Epidemiol, 37(7): 658-665.
5. Egger, M., Davey Smith, G., Schneider, M., & Minder, C. 1997. Bias in meta-analysis detected by a simple, graphical test. BMJ, 315(7109): 629-634.
6. Johnson, T., & Uk, S. 2012. Efficient calculation for multi-SNP genetic risk scores.
7. Palmer, T. M., Lawlor, D. A., Harbord, R. M., Sheehan, N. A., Tobias, J. H., Timpson, N. J., et al. 2012. Using multiple genetic variants as instrumental variables

for modifiable risk factors. Stat Methods Med Res, 21(3): 223-242.
8. Suurmond, R., van Rhee, H., & Hak, T. 2017. Introduction, comparison, and validation of Meta-Essentials: A free and simple tool for meta-analysis. Res Synth Methods, 8(4): 537-553.
9. Dai, J. Y., K. C. Chan, and L. Hsu. 2014. Testing concordance of instrumental variable effects in generalized linear models with application to Mendelian randomization. Statistics in medicine 33: 3986-4007.
10. Bowden, J., W. Spiller, M. F. Del Greco, N. Sheehan, J. Thompson, C. Minelli, and G. Davey Smith. 2018. Improving the visualization, interpretation and analysis of two-sample summary data Mendelian randomization via the Radial plot and Radial regression. International journal of epidemiology 47: 1264-1278.
